# Specific Probiotics for the Treatment of Pediatric Acute Gastroenteritis in India: A Systematic Review and Meta-Analysis

**DOI:** 10.1097/PG9.0000000000000079

**Published:** 2021-05-27

**Authors:** Lynne V. McFarland, Ramesh Srinivasan, Rajendra P. Setty, Sridhar Ganapathy, Ashish Bavdekar, Monjori Mitra, Bhaskar Raju, Neelam Mohan

**Affiliations:** From the * Department of Medicinal Chemistry, University of Washington, Seattle, WA; † Department of Gastroenterology, Apollo Hospitals, Jubilee Hills, Hyderabad, India; ‡ Department of Pediatric Gastroenterology, Hepatology and Nutrition, Pure Bliss Hospital, Panchkula, India; § Janani Children’s Hospital, Mumbai, India; ‖ Department of Pediatrics, K.E.M. Hospital, Pune, India; ¶ Department of Pediatrics, Institute of Child Health, Kolkata, India; # Department of Gastroenterology, Dr. Mehta’s Children’s Hospital, Chennai, India; ** Department of Pediatric Gastroenterology, Medanta Medicity, Gurgaon, India.

**Keywords:** diarrhea, *Saccharomyces boulardii*, *Lactobacillus rhamnosus*, rotavirus

## Abstract

Pediatric acute gastroenteritis (PAGE) is a significant cause of morbidity, mortality and healthcare costs in many countries, but differences in PAGE vary from country-to-country; thus, we limited our analysis to 1 country. Probiotics have been recommended as an adjunct to standard treatment, but the choice of probiotic is unclear. PubMed, Google Scholar, and reviews were searched from inception to May 2020 for randomized controlled trials (RCTs) in India using probiotics for a treatment for PAGE. Meta-analyses using subgroups of identical probiotic types (≥2 RCT/type) were conducted for primary outcomes (duration of diarrhea, cured by day 3, rapidity of response, and length of hospital stay). Twenty-two RCTs were included in the systematic review (N = 4059 participants) including 5 single-strained probiotics and 3 multi-strained mixtures. For the meta-analyses, 17 RCT (20 treatment arms) were included. *Saccharomyces boulardii* CNCM I-745 had the strongest effect on shortening the duration of diarrhea (standardized mean difference, –1.86 d; 95% confidence interval, –2.8 to –0.9), while both *Lactobacillus rhamnosus* GG and a mixture of 4 *Bacillus clausii* strains (O/C, SIN, N/R, T) significantly reduced the duration of diarrhea (–1.7 and –1.4 d, respectively). *S. boulardii* and *L. rhamnosus* GG significantly reduced hospital stays (−1.8 and −1.1 d, respectively), while *B. clausii* had no effect. The frequency of stools/day was significantly reduced by day 4 for *S. boulardii* and by day 5 for *L. rhamnosus* GG. In India, 2 types of probiotics (*S. boulardii* CNCM I-745 and *L. rhamnosus* GG) significantly shortened both the duration of diarrhea and hospitalization stays in pediatric patients with PAGE. While these 2 probiotic strains were safe and effective for children in India, further research is needed to confirm if other probiotic strains or mixtures may be effective.

## Abstract 2

Supplemental Digital Content is available in the text.

**What Is Known**

- Pediatric acute gastroenteritis (PAGE) is a significant cause of morbidity and mortality globally, especially in children under 5 years old and is more severe in developing countries.
- Most clinical trials studying the efficacy of probiotics for PAGE have been done in developed countries.
- The choice of an appropriate probiotic is strain-specific, but it is not known if efficacy differs depending upon study population’s country.

**What Is New**

- Only 3 types of probiotics had sufficient clinical trials (total 17 randomized controlled trials) in India to be included in a meta-analysis.
- Two probiotics (*Saccharomyces boulardii* CNCM I-745 and *Lactobacillus rhamnosus* GG) significantly reduced both the duration of diarrhea (1–2 d) and length of hospitalization (1–2 d) compared with controls in children living in India.

**Translational Impact**

- Two types of probiotics added to standard treatments for PAGE were found effective and safe in clinical trials done in India, but other probiotics required further trials.

## INTRODUCTION

Pediatric acute gastroenteritis (PAGE) is a leading cause of morbidity and mortality in children under 5 years old (1–3). Globally, 1.7 billion cases of PAGE occur each year, with 90% of cases occurring in developing countries (1,4). Deaths due to PAGE in children under 5 years were found to vary from country-to-country in a survey done in 2017: Africa (3%–13%), Asia (1%–9%), with the highest rates found in Syria (20%) (2). Factors influencing the incidence and severity of PAGE in different geographic areas include water sanitation, degree of malnourishment, diet, lifestyle factors, and type of diarrhea etiologies (3,5). It is difficult to account for all these factors when assessing efficacy of new treatments; thus, we focused our review on trials done in 1 country (India).

In India, improvements in water quality and use of oral rehydration therapy (ORT) has resulted in a 40% reduction of PAGE-associated mortality since 2001, but PAGE continues to be a significant cause of morbidity, mortality, increased hospital admissions, and economic burden (4–8). Etiologies of PAGE in India may also differ from European countries. In Europe, the most common etiologies were found to be *Campylobacter* or *Salmonella* (9). In 1 study of outpatient children under 5 years in Odisha, India, the most common etiologies for PAGE were *E. coli* (30%), rotavirus (26%), or *Shigella* (24%), and concurrent infection with multiple etiologies was frequent (34%) (10).

Shifts in the normally protective intestinal microbiome are found when diarrhea is present (11,12) and use of some strains of living probiotics has been found to be useful in restoring the normal microbiome (13,14). The choice of an appropriate probiotic is challenging due to the diverse types of probiotics available, inconsistent reports of efficacies and strength of available evidence (15). As the efficacy of probiotics has been demonstrated to be both disease-specific and strain-specific, it is important to assess efficacy only within the identical type of probiotic strain or strains within multi-strain mixtures for 1 type of disease (16,17). Many reviews and meta-analyses have either incorrectly pooled different strains of probiotics together (18) or limited their review to 1 strain of probiotic, but then pooled studies done in developed and developing countries (19–23). The pooled estimates of efficacy may be biased due to heterogeneity related to different probiotic strains or by different factors (microbiome profiles, socioeconomic factors, etc.) related to geographic area. The recent American Gastroenterology Association guidelines recommended against probiotics for PAGE in the United States and Canada, as most randomized controlled trials (RCTs) were done outside of these 2 countries, thus highlighting the importance of country-to-country variations (24).

This review focuses on randomized clinical trials only done in 1 country (India) in an effort to reduce these diverse sources of heterogeneity. Our aim is to determine which types of probiotics are safe and clinically effective for management of PAGE in the Indian population.

## METHODS

Primary outcomes included differences in the mean duration of acute diarrhea from enrollment to resolution or the end of the study in subgroups of probiotics with the same strain or mixture of strains compared with the duration in the control groups. The other primary outcomes were the frequency of children with diarrhea resolution by day 3 or 5 (“cured”) of treatment in subgroups of probiotics with the same strain or mixture of strains compared with children in the control group and the rapidity of response (the mean number of stools/day from day 1, to day 3, 4, or 5) for the probiotic and control groups. Secondary outcomes included differences in length of hospitalization and differences in the frequency of adverse events between the study groups.

Standard search strategy, inclusion/exclusion criteria, data extraction, statistical methods, and assessment procedures were followed and described (see Text, Supplemental Digital Content 1, Methods, http://links.lww.com/PG9/A30) (25–30).

## RESULTS

### Search Results

The literature search yielded 235 articles on probiotic use for the treatment of PAGE (see Figure, Supplemental Digital Content 1, PRISMA Flow-Chart, http://links.lww.com/PG9/A32; Table, Supplemental Digital Content 1, PRISMA Checklist, http://links.lww.com/PG9/A40). Reasons for exclusion (n = 213 studies) are provided elsewhere (see Text, Supplemental Digital Content 2, Results, http://links.lww.com/PG9/A31). Twelve trials done in India but not meeting inclusion criteria were excluded (see Table, Supplemental Digital Content 2, Excluded Trials, http://links.lww.com/PG9/A41) (31–42).

Studies included in the systematic review (n = 22 RCTs, N = 4059 participants) included 5 single-strain probiotics and 3 multi-strained mixtures (Table 1) (43–64). Five types of probiotics were excluded from the meta-analysis (60–64), as they lacked at least 1 confirmatory trial, resulting in 17 RCTs for the meta-analysis (20 treatment arms) for 3 probiotics: *Saccharomyces boulardii* CNCM I-745 (9 RCTs), *Lactobacillus rhamnosus* GG (ATCC 53103) (6 RCT, 7 arms), and a 4-strain mixture of *Bacillus clausii* O/C, SIN, N/R, T (4 RCTs). Two trials compared 2 different probiotics to a control group (43,51) and 1 trial had 2 treatment arms with different doses of the probiotic (55).

**TABLE 1.** Probiotic and control intervention characteristics of 22 randomized controlled trials (25 treatment arms) in India for the treatment of Pediatric Acute Gastroenteritis

Probiotic	Daily dose (CFU/d)	Formulation	Duration (d)	Type of control	ORT given	Zinc	Initiation Time (d)	Overall risk of bias	References
*Saccharomyces boulardii* CNCM I-745	1 × 10^10^	NR	6	Open	Yes	NR	1.2 ± 0.6	High	Bhat et al (43)
*S. boulardii* CNCM I-745	1 × 10^10^	Sachet	5	Open	Yes	Yes	<2	Low	Burande and Burande (44)
*S. boulardii* CNCM I-745	1 × 10^10^	Sachet	5	Placebo	NR	NR	3 ± 1	Low	Das et al (45)
*S. boulardii* CNCM I-745	1 × 10^10^	Sachet	5	Open	Yes	Yes	NR	High	Dash et al (46)
*S. boulardii* CNCM I-745	1 × 10^10^	Sachet	3	Open	PRN	NR	NR	High	Kumar et al (47)
*S. boulardii* CNCM I-745	1 × 10^10^	Powder	5	Placebo	Yes	Yes	0.9 ± 0.8	Low	Riaz et al (48)
*S. boulardii* CNCM I-745	1 × 10^10^	Powder	5	Open	Yes	NR	NR	High	Sirsat and Sankpal (49)
*S. boulardii* CNCM I-745	1 × 10^10^	NR	5	Placebo	Yes	No	NR	Low	Vandenplas et al (50)
*S. boulardii* CNCM I-745	1 × 10^10^	Sachet	NR	Open	Yes	Yes	NR	Low	Vidjeadevan et al (51)
*Lactobacillus rhamnosus* GG	1 × 10^10^	Capsule	5	Open	Yes	Yes	2.2 ± 1.3	Low	Aggarwal et al (52)
*L. rhamnosus* GG	1 × 10^10^	NR	5	Open	NR	NR	2.5 ± 1.0	High	Agarwal (53)
*L. rhamnosus* GG	1.2 × 10^8^	Liquid	7	Placebo	Yes	No	NR	Low	Basu et al (54)
*L. rhamnosus* GG-low dose	2 × 10^10^	Liquid	7	Placebo	Yes	No	NR	Low	Basu et al (55)
*L. rhamnosus* GG-high dose	2 × 10^12^	Liquid	7	Placebo	Yes	No	NR	Low	Basu et al (55)
*L. rhamnosus* GG	1 × 10^6^–10^9^	Capsule	3–10	Placebo	NR	No	1.9 ± NR	Low	Misra et al (56)
*L. rhamnosus* GG	1 × 10^10^	Capsule	28	Placebo	NR	NR	4 ± NR	Low	Sindhu et al (57)
*Bacillus clausii* O/C, SIN, N/R, T	4 × 10^9^	Spores	6	Open	Yes	NR	1.2 ± 0.6	High	Bhat et al (43)
*B. clausii* O/C, SIN, N/R, T	4 × 10^9^	Liquid	5	Open	Yes	Yes	NR	High	Lahiri et al (58)
*B. clausii* O/C, SIN, N/R, T	4 × 10^9^	Liquid	5	Open	Yes	Yes	NR	High	Lahiri et al (59)
*B. clausii* O/C, SIN, N/R, T	2 × 10^9^	Spores	NR	Open	Yes	Yes	NR	Low	Vidjeadevan et al (51)
Bifilac (4 strains)	2.5 × 10^8^	Sachets	14	Placebo	Yes	NR	<3	Low	Narayanappa (60)
*B. clausii* UBBC-07	4 × 10^9^	Liquid	5	Placebo	Yes	NR	NR	Low	Sudha et al (61)
*L. casei* DN114001	1 × 10^10^	NR	5	Open	NR	No	NR	Low	Agarwal and Bhasin (62)
*L. sporogenes*	2.4 × 10^9^	Tablets	5	Placebo	Yes	No	<3	Low	Dutta et al (63)
8 strain mixture	2.4 × 10^11^	Liquid	4	Placebo	Yes	NR	<3	Low	Dubey et al (64)

*L. rhamnosus* GG (ATCC 53103); Bifilac: 4 strain mixture: *Clostridium butyricum*, *Bacillus mesentericus*, *Streptococcus faecalis*, and *L. sporogenes*, strains not reported, from author correspondence; 8 strain mixture: *L. plantarum* DSM24730, *S. thermophilus* DSM24731, *Bifidobacterium breve* DSM24732, *L. delbrueckii ssp. bulgaricus* DSM24733*, L. paracasei* DSM24734, *L. acidophilus* DSM24735, *B. longum* DSM24736, and *B. infantis* DSM24737. CFU/d = colony-forming units per day; Initiation Time = mean time from onset of diarrhea to initiation of probiotic; NR = not reported in article; ORT = oral rehydration therapy; PRN = given as needed.

### Trial Characteristics

The study participant characteristics and safety data are described supplementary files (see Text, Supplemental Digital Content 2, Results, http://links.lww.com/PG9/A31 and in Table, Supplemental Digital Content 3, Study Population, http://links.lww.com/PG9/A42). Most trials provided complete descriptions of the probiotic intervention (Table 1), but 10 trials did not provide the manufacturer or brand name (see Table, Supplemental Digital Content 4, Probiotic Description, http://links.lww.com/PG9/A43).

Of the 17 RCTs with ≥2 RCTs/probiotic type, 59% had an overall low risk of bias, while 41% had an overall high risk of bias (Table 1). Most of the trials had a low risk of bias for randomization method (59%), attrition (88%), open/placebo controls (53%), and reporting post hoc outcomes (94%) (see Figure, Supplemental Digital Content 2, Risk of Bias, http://links.lww.com/PG9/A33). Ten (59%) of the 17 RCTs were double-blinded using placebos, while 7 (41%) used open controls (standard treatments only). There was no significant impact of blinding on the estimated treatment effects; thus, these types of controls were combined. For example, in trials with *S. boulardii*, the mean duration of diarrhea for unblinded (open) studies was 2.3 days for *S. boulardii* versus 3.6 days for open controls and comparable data were observed (2.7 d for *S. boulardii* versus 3.6 d for placebo controls), and results were similar for *L. rhamnosus* GG trials, while all *B. clausii* trials used open controls. Trials ranked as high risk typically did not describe the method of randomization, whether the study allocation was blinded, or did not report if the outcome was determined by blinded study staff. The risk of bias had an inconsistent impact on outcome measures by the type of probiotic: trials with *S. boulardii* showed the greatest reduction in duration of diarrhea in low bias risk trials (see Table Supplemental Digital Content 5, Subgroup Analysis, http://links.lww.com/PG9/A44), while the greatest reduction was seen in high-risk trials with *L. rhamnosus* GG and 3 of 4 trials with *B. clausii* mix were of high risk.

### Meta-Analysis of Probiotic Efficacy

#### Duration of Diarrhea

**FIGURE 1.**
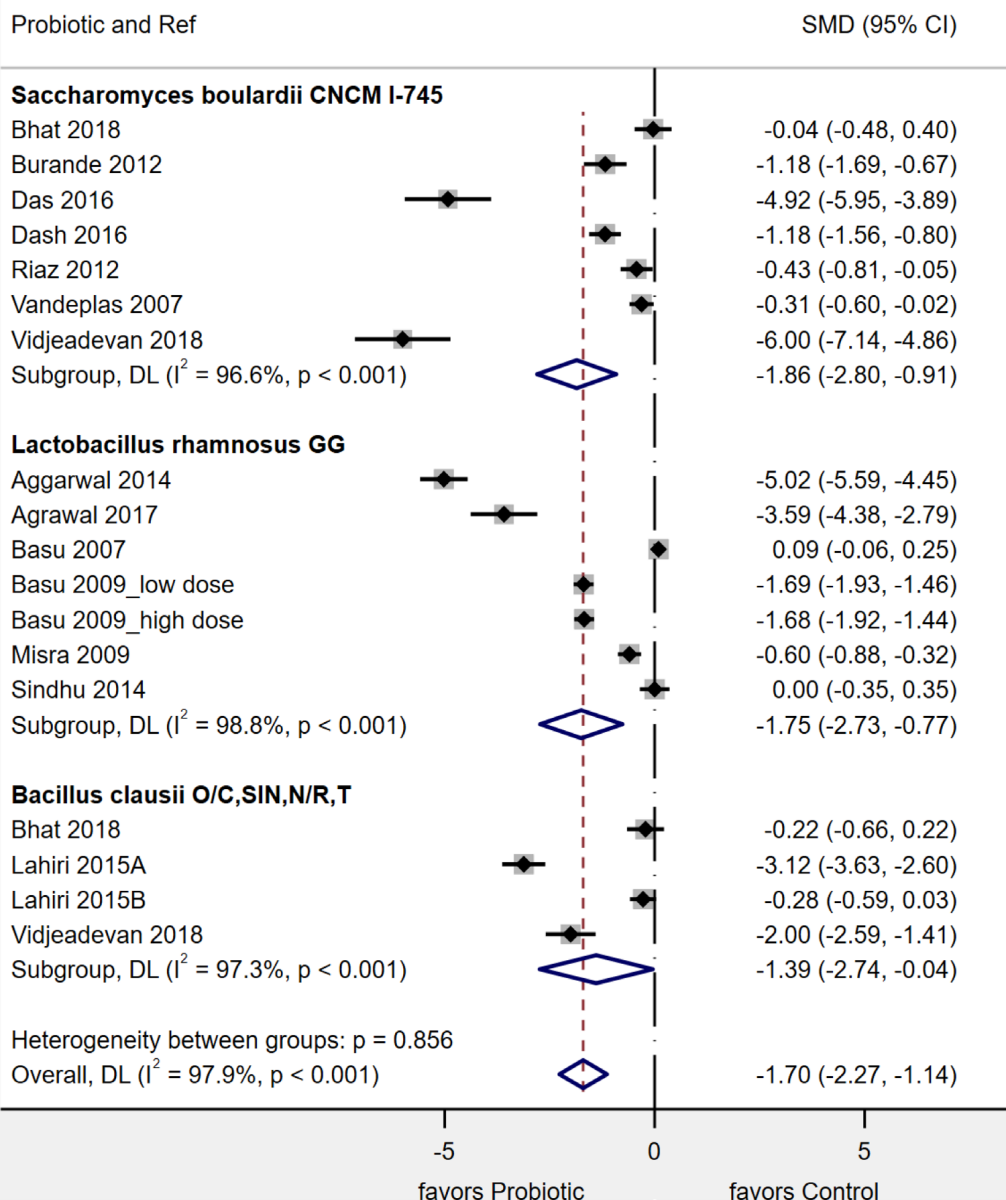
Forest plot of 17 randomized controlled trials done in India for the mean reduction in the duration of Pediatric Acute Gastroenteritis (d) with 3 different probiotics. CI = confidence interval; DL = DerSimonian-Laird estimate of between study variance; SMD = standardized mean difference.

Of the 17 RCTs (20 treatment arms), most (15, 88%) reported the mean duration of diarrhea as the primary outcome (Table 2). As shown in Figure 1, 3 types of probiotics significantly reduced the mean duration PAGE. *S. boulardii* CNCM I-745 had the strongest effect on the reduction of diarrhea (standardized mean difference [SMD], –1.86 d; 95% confidence interval [CI], –2.8 to –0.91 d; *P* < 0.001, *I*^2^ = 96.6%). *L. rhamnosus* GG also significantly reduced the duration of diarrhea, but to a lesser extent (SMD, –1.75 d; 95% CI, –2.73 to –0.77 d; *P* = 0.001; *I*^2^ = 98.8%). The 4-strain mixture of *B. clausii* O/C, SIN, N/R, T also significantly reduced the duration of diarrhea (SMD, –1.39; 95% CI, –2.74 to –0.04; *P* = 0.04; *I*^2^ = 97.3%). There was significant heterogeneity found among these studies (I2 = 97%–99%) and publication bias was noted (Egger’s t = –3.3; *P* = 0.005), as shown in the funnel plot (see Figure, Supplemental Digital Content 3, Publication Bias, http://links.lww.com/PG9/A34). Sensitivity analysis found no one individual trial had undue influence on the pooled outcome of the mean duration of diarrhea.

Subgroup analyses found several factors influenced the effect of probiotics on the mean duration of diarrhea (see Table, Supplemental Digital Content 5, Subgroups, http://links.lww.com/PG9/A44). In 4 RCTs, when *S. boulardii* CNCM I-745 was added to ORT and zinc, there was a significant reduction of the duration of diarrhea (SMD, –2.05 d; 95 CI, –3.4 to –0.75 d; *P* = 0.002). In 1 study, no zinc was given (50) and 4 RCTs did not report if zinc was given or not (43, 45, 47, 49). Trials using *S. boulardii* with a low risk of bias showed a greater reduction in diarrhea duration, but the difference was not significant (Cochrane’s Q = 2.1; *P* = 0.15). No other subgroups significantly influenced the efficacy of *S. boulardii* CNCM I-745.

Subgroups resulting in a significant reduction in heterogeneity for *L. rhamnosus* GG trials included: use of zinc (Cochrane’s Q = 38.0; *P* < 0.001), risk of bias (Cochrane’s Q = 5.8; *P* = 0.02), unblinded controls (Cochrane’s Q = 18.2; *P* = 0.001), and higher (≥10^10^ colony-forming units per day [CFU]/d) dose (Cochrane’s Q = 5.24; *P* = 0.02). For 2 subgroups (zinc and risk of bias), only 1 trial used zinc (52) and only 1 trial had a high risk of bias (53), so conclusions should not be made for these factors. For factors (daily dose and blinded study design), more robust conclusions can be made as there were ≥2 trials/subgroup. For *L. rhamnosus* GG trials, daily doses ≥10^10^/d resulted in a significantly greater reduction in days of diarrhea (SMD, –2.4 d; 95% CI, –3.6 to –1.1; *P* < 0.001) compared with lower doses (SMD, –0.2 d; 95% CI, –0.92 to 0.43; *P* = 0.48). For trials comparing placebo to *L. rhamnosus* GG, the mean reduction was not significant (SMD, –0.78; 95% CI, –1.61 to 0.05; *P* = 0.07), while use of open controls resulted in a significant reduction of diarrhea (SMD, –4.33; 95% CI, –5.73 to –2.93; *P* < 0.001).

Subgroup analysis for *B. clausii* trials was limited, as all trials were in inpatients and used open controls and all 4 trials gave ORT to the subjects. Zinc was also given in 3 trials but not reported in 1 trial (43). Subgroups were not significantly different: daily dose (Cochrane’s Q = 0.63; *P* = 0.43) and low versus high risk of bias (Cochrane’s Q = 0.63; *P* = 0.43).

Other subgroup analyses found factors that did not significantly impact outcome measures for PAGE for any of the 3 probiotics included: probiotic formulation, probiotic initiation times, use of ORT, rural versus urban settings, or funding sources (see Table, Supplemental Digital Content 5, Subgroups, http://links.lww.com/PG9/A44).

#### Duration of Diarrhea in Rotavirus-Positive Children

Only 2 types of probiotics had sufficient trials to assess rotaviral diarrhea (see Table, Supplemental Digital Content 6, Rotaviral Diarrhea, http://links.lww.com/PG9/A45). Two RCTs using *S. boulardii* reported outcomes for rotavirus-positive children, but different outcome measures were used. Das et al (45) found *S. boulardii* significantly reduced diarrhea duration by –1.2 ± 0.1 days and Sirsat and Sankpal (49) found 25% more children were cured by day 3 if given *S. boulardii* compared with controls. *L. rhamnosus* GG reported mean duration of diarrhea in 3 trials of rotavirus-positive patients (52,53,57). The reduction in diarrheal days in rotavirus-positive children was not significant for *L. rhamnosus* GG (SMD, –3.11 d; 95% CI, –9.29 to 3.1 d; *P* = 0.32; *I*^2^ = 98%). The 4 RCTs with *B. clausii* mixture did not report data by diarrheal etiology.

#### Cured by Day 3

Only *S. boulardii* CNCM I-745 had sufficient trials to explore this outcome, with 4 trials reporting the cure rates by day 3 (47, 49–51). There was a trend (*P* = 0.054) of cure by day 3 (relative risk, 1.55; 95% CI, 0.90–2.41; *I*^2^ = 90%; see Figure, Supplemental Digital Content 4, Cured by Day 3, http://links.lww.com/PG9/A35). Sensitivity analysis found no one individual trial had undue influence on the pooled outcome. The relative risk was not significantly influenced when only 2 trials of low risk of bias were included nor by the degree of blinding, or by dose, or by the addition of zinc.

#### Rapidity of Response (Stool Frequency/Day Over Time)

The rapidity of a response was different by the type of probiotic, but not all trials provided data for this outcome (see Table, Supplemental Digital Content 7, Rapidity of Response, http://links.lww.com/PG9/A46). In 3 RCTs with *S. boulardii* CNCM I-745 (43, 46, 48), the number of stools/day were equivalent at day 1 and day 3, but by day 4, the mean number of stools was significantly fewer for *S. boulardii* compared with controls (SMD, –0.61 stools/day; 95% CI, –1.06 to –0.17; *P* = 0.007), as shown in Figure (Supplemental Digital Content 5, *S. boulardii* Response, http://links.lww.com/PG9/A36), although only 1 trial measured this outcome on day 4. In 2 RCTs (3 study arms) with *L. rhamnosus* GG (54,55), a significant difference in stools/day was not observed until day 5 with SMD = –1.1 stools/day; 95% CI, –2.11 to –0.08; *P* = 0.03 (see Figure, Supplemental Digital Content 6, *L. rhamnosus* Response, http://links.lww.com/PG9/A37). In 2 RCTs with *B. clausii* mixture, the number of stools/day was equivalent to controls for days 1, 3, and 5 (see Figure, Supplemental Digital Content 7, *B. clausii* Mix Response, http://links.lww.com/PG9/A38).

#### Length of Hospital Stay

**FIGURE 2.**
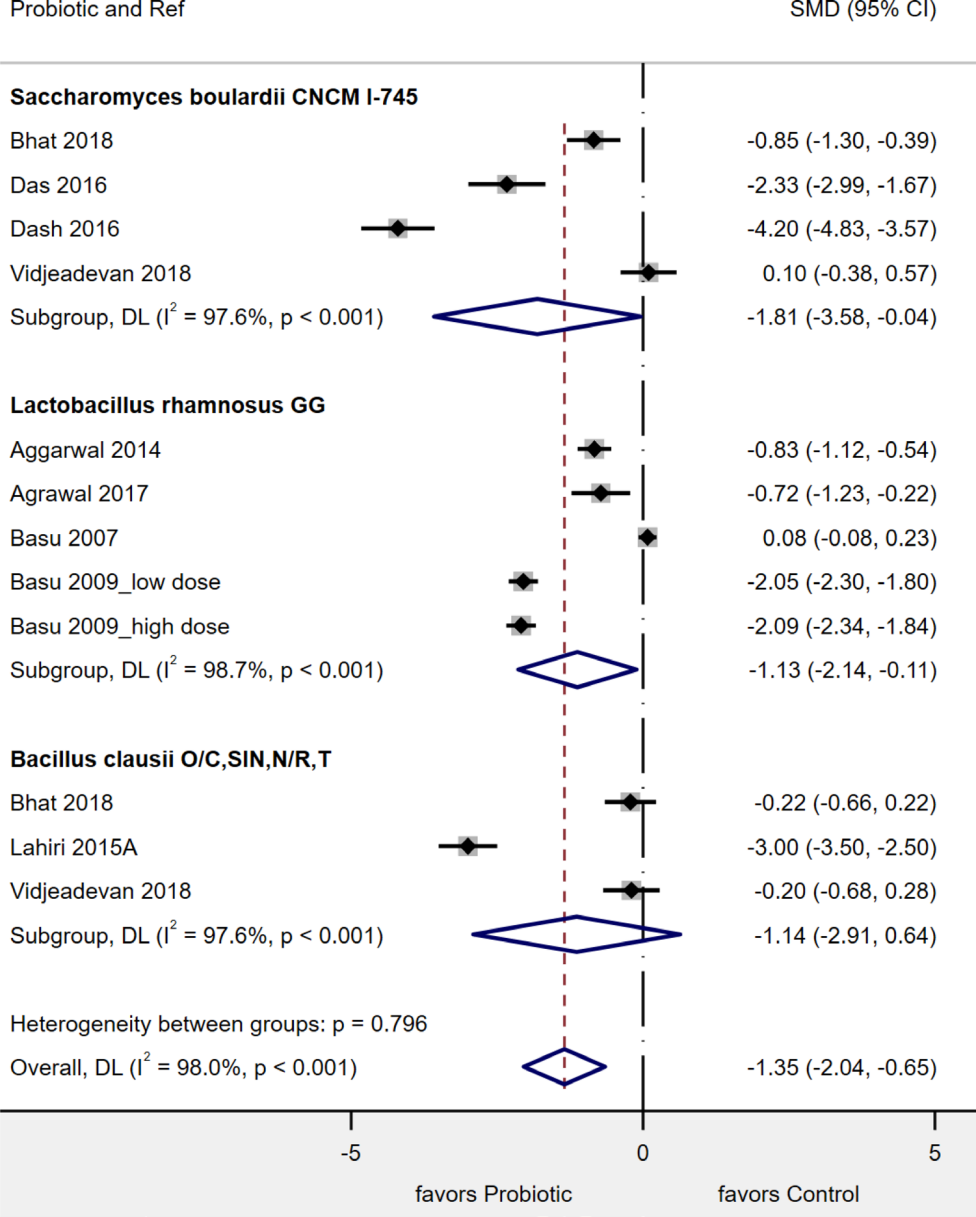
Forest plot of 11 randomized controlled trials in India for the mean reduction of hospitalization (length of stay [d]) with 3 different types of probiotics. CI = confidence interval; DL = DerSimonian-Laird estimate of between study variance; SMD = standardized mean difference.

Of the 12 RCTs that enrolled inpatient children, 9 trials provided data on the mean length of hospitalization stay (LOS) for the probiotic compared with the control group (see Table, Supplemental Digital Content 8, LOS, http://links.lww.com/PG9/A47). As shown in Figure 2, 2 types of probiotics significantly reduced the mean duration of hospitalization. *S. boulardii* CNCM I-745 had the strongest effect on the reduction of mean LOS (SMD = –1.81 d; 95% CI, –3.58 to –0.04 d; *P* = 0.04; *I*^2^ = 98%). *L. rhamnosus* GG also significantly reduced the mean LOS (SMD, –1.13 d; 95% CI, –2.14 to –0.11 d; *P* = 0.03; *I*^2^ = 99%). The 4-strain mixture of *B. clausii* O/C, SIN, N/R, T did not significantly reduce the LOS (SMD, –1.14; 95% CI, –2.91 to 0.64; *P* = 0.21; *I*^2^ = 98%). There was significant heterogeneity found among these studies (97%–98%), but no significant publication bias was noted (Egger’s *P* = 0.16).

#### Safety

Of the 25 study arms, 11 (44%) did not collect any adverse reaction data (Table 2), but 14 (56%) did collect and report adverse event data. Of the 14 with safety data, 10 (71%) reported no adverse events were observed during the study, while 4 (29%) reported at least 1 child with an adverse event, but the frequency was not significantly different for probiotic compared with control groups. None of the different probiotic types were associated with significant adverse events.

**TABLE 2.** Main outcomes for probiotic and control groups from 22 randomized controlled trials (25 treatment arms) in India for the treatment of Pediatric Acute Gastroenteritis

Probiotic	Probiotic		Control		Probiotic	Control	Probiotic	Control	References
	N	Duration of diarrhea (mean d ± SD)	N	Duration of diarrhea (mean d ± SD)	Cured by day 3, n (%)	Cured by day 3, n (%)	AE (%)	AE (%)	
*Saccharomyces boulardii* CNCM I-745	40	1.7 ± 0.4	40	2.4 ± 1.1	NR	NR	NR	NR	Bhat et al (43)
*S. boulardii* CNCM I-745	35	3.4 ± 1.4	35	5.5 ± 2.1	NR	NR	NR	NR	Burande and Burande (44)
*S. boulardii* CNCM I-745	30	2.5 ± 0.2*	30	3.7 ± 0.3*	NR	NR	0	0	Das et al (45)
*S. boulardii* CNCM I-745	64	1.1 ± 0.5*	62	2.0 ± 1.0*	NR	NR	0	0	Dash et al (46)
*S. boulardii* CNCM I-745	50	Unclear	50	Unclear	15 (30)	8 (16)	0*	0*	Kumar et al (47)
*S. boulardii* CNCM I-745	54	2.2 ± 2.0	54	2.7 ± 1.3	NR	NR	0	0	Riaz et al (48)
*S. boulardii* CNCM I-745	145	NR	145	NR	97 (67)	72 (49)	NR	NR	Sirsat et al (49)
*S. boulardii* CNCM I-745	93	2.2 ± 1.6	95	2.8 ± 2.2	90 (97)	85 (90)	0	0	Vandenplas et al (50)
*S. boulardii* CNCM I-745	34	3.0 ± 0.2*	32	4.5 ± 0.2*	20 (59)	5 (16)	NR	NR	Vidjeadevan et al (51)
*Lactobacillus rhamnosus* GG	100	2.5 ± 0.1	100	3.2 ± 0.1	NR	NR	0	0	Aggarwal et al (52)
*L. rhamnosus* GG	32	2.5 ± 1.9	33	3.2 ± 1.9	NR	NR	0	0	Agarwal (53)
*L. rhamnosus* GG	323	6.8 ± 2.1	323	6.6 ± 2.3	NR	NR	1	2	Basu et al (54)
*L. rhamnosus* GG-low dose	188	5.0 ± 1.3	185	7.2 ± 1.3	NR	NR	2	4	Basu et al (55)
*L. rhamnosus GG*-high dose	186	5.1 ± 1.2	185	7.2 ± 1.3	NR	NR	4	4	Basu et al (55)
*L. rhamnosus* GG	105	2.9 ± 0.5*	105	3.2 ± 0.5*	NR	NR	NR	NR	Misra et al (56)
*L. rhamnosus* GG	65	4.0 ± 2.2*	59	4.0 ± 2.2*	NR	NR	6	2	Sindhu et al (57)
*Bacillus clausii* O/C, SIN, N/R, T	40	2.2 ± 0.7	40	2.4 ± 1.1	NR	NR	NR	NR	Bhat et al (43)
*B. clausii* O/C, SIN, N/R, T	69	0.9 ± 0.4*	62	1.96 ± 0.4*	NR	NR	NR	NR	Lahiri et al (58)
*B. clausii* O/C, SIN, N/R, T	80	0.9 ± 1.7*	80	1.4 ± 1.7*	NR	NR	NR	NR	Lahiri et al (59)
*B. clausii* O/C, SIN, N/R, T	33	4.0 ± 0.2*	32	4.5 ± 0.2*	15 (45)	5 (16)	NR	NR	Vidjeadevan et al (51)
Bifilac (4 strains)	40	4.3 ± 1.2	40	5.4 ± 1.7	NR	NR	0	0	Narayanappa (60)
*B. clausii* UBBC-07	59	3.1 ± 0.6	60	3.4 ± 0.6	41 (69.5)	35 (58.3)	NR	NR	Sudha et al (61)
*L. casei* DN114001	32	1.5 ± 0.5	33	2.1 ± 0.7	NR	NR	NR	NR	Agarwal and Bhasin (62)
*L. sporogenes*	78	1.4 ± 0.8	70	1.5 ± 0.9	70 (89.7)	58 (82.9)	0	0	Dutta et al (63)
8 strain mixture	113	NR	111	NR	101 (89.4)	44 (39.6)	0	0	Dubey et al (64)

*Data supplied by author; or estimated SD; Bifilac: 4 strain mixture: *Clostridium butyricum*, *Bacillus mesentericus*, *Streptococcus faecalis*, and *L. sporogenes*, strains not reported, or; 8 strain mixture: *L. plantarum* DSM24730, *S. thermophilus* DSM24731, *Bifidobacterium breve* DSM24732*, L. delbrueckii ssp. bulgaricus* DSM24733, *L. paracasei* DSM24734, *L. acidophilus* DSM24735*, B. longum* DSM24736, and *B. infantis* DSM24737. AE = adverse event; NR = not reported in article; SD = standard deviation.

## DISCUSSION

We conducted a systematic review and meta-analysis of 22 RCTs (with 4059 participants) to estimate the efficacy of probiotics available in India for the treatment of PAGE. Our study is a unique study focusing on clinical trials in 1 developing country (India) in an effort to limit geographic and nutritional factors that may influence rates of pediatric acute diarrhea. We included 17 RCTs in our meta-analysis of 3 different types of probiotics. We found differences in efficacy by type of probiotic: *S. boulardii* CNCM I-745 had the greatest reduction in mean days of diarrhea and reduction in LOS for hospitalized children and had the most rapid response by number of stools/day (reduced by day 4), whereas *L. rhamnosus* GG did not show a significant reduction in stool frequency until day 5. Trials using *B. clausii* did not show a significant difference by day 5. Most trials followed World Health Organization recommendations to use ORT and zinc in India or other countries where zinc deficiency is found (1), but only 41% used zinc as recommended. Zinc was not added to ORT in 24% of the RCTs and 35% of the RCTs failed to report if zinc was added or not.

Guidelines for which probiotics may be recommended for PAGE have been inconsistent. The 2020 American Gastroenterology Association guidelines did not recommend probiotics, citing most trials have been done in other countries besides the United States or Canada (24). In Europe, a recent update of the European Society of Paediatric Gastroenterology, Hepatology and Nutrition had weak recommendations for *S. boulardii* (based on 22 RCTs), *L. rhamnosus* GG (16 RCTs), and 2 other probiotics with *L. reuteri* DSM17938 (6 RCTs), but recommended against the use of *B. clausii* mix or a mix of *L. helveticus* R52 and *L. rhamnosus* R11 (65).

We found 7 meta-analyses previously published on probiotics and treatment of PAGE (19–23, 65, 66) and all combined results from trials done in different countries. An updated Cochrane meta-analysis included 79 RCTs in children and adults with acute gastroenteritis in different countries and found a mean of 1-day reduction in diarrhea for each of the following: *S. boulardii* (11 RCTs), *L. rhamnosus* GG (14 RCTs), and *L. reuteri* DSM 17938 (6 RCTs), but did not present pediatric trials separately by country (66).

Two meta-analyses of *S. boulardii* trials found significant efficacy for PAGE (20, 23). Feizizadeh et al (20) included 22 RCTs done in 12 different countries and found *S. boulardii* reduced the duration of diarrhea by –0.82 days. Szajewska et al (23) included 23 RCTs in her meta-analysis of *S. boulardii* trials from 11 different countries and also found a significant reduction in duration of diarrhea (SMD, –1.1 d; 95% CI, –1.3 to –0.8 d) and shorter LOS for inpatients (8 RCT; SMD, 0.85 d; 95% CI, –1.3 to –0.3 d) and in rotaviral positive patients. In general, our results of trials limited to those done in India agreed with results found in other countries, showing probiotics may be effective in varied populations. In our meta-analysis of 7 RCTs done in India using *S. boulardii* CNCM I-745, we found a greater reduction in the duration of diarrhea (SMD, –1.86 d; *P* < 0.001) and shorter hospitalization stays (4 RCTs; SMD, –1.81 d; *P* < 0.001) and significant efficacy in rotaviral diarrhea. Subgroup analysis determined factors not impacting the efficacy of *S. boulardii* included: daily probiotic dose, in/outpatient status, degree of blinding, or type of controls used. The time of probiotic initiation was not a factor in the Indian trials, as *S. boulardii* was started within 3 days of diarrheal onset in all reported trials. No dose-response was observed, as all trials used the same dose (1 × 10^10^ CFU/d).

Two previous meta-analyses were done including trials only using *L. rhamnosus* GG and both found a significant mean reduction in duration of diarrhea: in 15 RCTs (SMD, –0.85 d; 95% CI, –1.15 to –0.56) (22) and in 19 RCTs (SMD, –1.0 d; 95% CI, –1.5 to –0.5) (67). Szajewska et al (22) reported *L. rhamnosus* GG was twice as effective in European countries compared with a mix of non-European countries (South East Asia, South America, United States, and Australia). A meta-analysis by Li et al (67) found doses of *L. rhamnosus* GG less than 10^10^ CFU/d, delayed probiotic initiation (>3 d) and non-Asian/non-European countries resulted in a loss of efficacy for duration of diarrhea by *L. rhamnosus* GG. Li et al (67) also found *L. rhamnosus* GG was more effective in rotaviral diarrhea for shortening diarrheal duration (7 RCTs; SMD, –1.3 d; *P* < 0.001) and reduced LOS by 1.3 days. Our results agree with these 2 meta-analyses done in different countries. In our meta-analysis of 6 RCTs in India, *L. rhamnosus* GG appeared to be slightly more effective in reducing the duration of diarrhea (SMD, −1.75 d; *P* < 0.001) compared with the mixed group of different countries in the previous 2 meta-analyses. In our meta-analysis, *L. rhamnosus* GG also reduced the mean LOS in Indian inpatients (4 RCTs; SMD, –1.13 d; *P* < 0.001), but not as great as an extent as the previous 2 meta-analyses. In the Indian trials reported initiation times, *L. rhamnosus* GG was started within 4 days of the onset of diarrhea. Our meta-analysis also confirmed a sufficiently high dose (1 × 10^10^ CFU/d) was needed for *L. rhamnosus* GG to be significantly effective. It is also interesting that the same strain was not found to be effective in another country. A large RCT involving 971 children with PAGE admitted to 10 pediatric emergency departments across the United States failed to find efficacy for *L. rhamnosus* GG (68).

A meta-analysis by Ianiro et al (21) included 6 RCTs using *B. clausii* probiotics and found a significant reduction in the duration of diarrhea (SMD, –0.4 d; 95% CI, –0.69 to –0.07; *P* = 0.02) and a reduction in LOS (3 RCTs; SMD, –0.85 d; 95% CI, –1.6 to –0.15; *P* = 0.03), but did not conduct any subgroup analyses. In a recent review of probiotics for the management of acute gastroenteritis in children, this Bacillus mixture was not recommended to treat PAGE (65). In our meta-analysis, the 4-strain mixture of *B. clausii* appears was effective for the reduction of diarrheal duration when trials are done in India (SMD, –1.4 d; *P* < 0.001), but the reduction in LOS was not significantly reduced by the *B. clausii* mix. Two reasons why our results may differ from the previous reviews is that we only included RCTs done in India and, second, we had rigorous inclusion criteria that excluded unpublished or duplicative trials. These excluded trials typically had nonsignificant efficacy findings. An advantage of using living probiotics is that they may possess one or more multiple mechanisms of action, which may explain why specific probiotics are effective against different etiologies of PAGE (69, 70).

Strengths of our study included an exhaustive search of all RCTs in 1 country, including gray literature and meeting abstracts. The inclusion criteria were rigorous, in that only trials with well-described probiotic treatments (strains, daily dose, and formulations) were included and only those probiotics with at least 2 RCTs were included. Most trials used a standard definition of diarrhea (≥3 loose-watery stools/day) for the inclusion criteria. However, since there is no consensus for a standard outcome measure, the trials differed in the definition of diarrheal outcome (see Table, Supplemental Digital Content 4, Probiotic Description and Definitions, http://links.lww.com/PG9/A43) and types of outcomes.

Limitations were related to trials using different outcome measures, as we could only compare efficacy when common outcomes were used. In addition, probiotics with just a single RCTs could not be assessed and the proof of efficacy for some probiotic strains must wait until confirmatory trials are published. Factors that may be sources of heterogeneity were not reported in all trials. Reasons for heterogeneity may include differences in clinical baseline characteristics (such as age, degree of malnourishment, urban/rural settings, inpatient/outpatient, breast-fed versus formula-fed infants, etc.) or may due to differences in study design (degree of blinding, attrition, study quality, study size, adjunctive therapies given, etc.) Important confounder factors and influences were often not described in papers (diet, environmental factors, malnourishment status, etiologies of diarrhea, basic health status, mortality data, frequency of breast-feeding versus formula-fed, etc.). We were able to conduct subgroup analysis on most of these factors, but most did not significantly reduce heterogeneity. Daily doses less than 10^10^ CFU/d were not effective for *L. rhamnosus* GG, while trials with *S. boulardii* all used 2 × 10^10^ CFU/d and *B. clausii* used a lower dose (10^9^ CFU/d). Trials differed in quality of reporting (method of randomization not reported, allocation blinding not reported, etc.). About half (48%) used placebo, but 52% of the trials were not double-blinded. However, subgroup analysis by study design bias did not find this influenced efficacy measures. This confirms the finding by Moustgaard et al (71) who reviewed 142 meta-analyses and found no significant effect of blinding or placebo use on estimated treatment effects. When trials were grouped by low versus high risk of bias, trials with low risk had better efficacy with *S. boulardii* (see Figure, Supplemental Digital Content 8, Risk of Bias, http://links.lww.com/PG9/A39), while the interpretation of high risk is difficult for *L. rhamnosus* GG, as there is only 1 trial with high risk. This holds true for *B. clausii* mix trials, as only one trial was of low risk. The type of funding was also assessed (23% by academic grant, 9% funded by Pharma companies, 23% were unfunded) but 45% did not report funding sources. The type of funding did not appear to significantly influence efficacy outcomes.

### Generalizability and Future Studies

As the included trials were based upon study patients who lived in India, the results may not be extrapolated to probiotic efficacy in developed countries where the level of water sanitation and hygiene differ and the frequency of breast-feeding is typically lower. In addition, microbiome profiles and etiologies of diarrhea differ geographically and how this may influence probiotic efficacy is unknown. Implications for clinical practice and policy may include a more rational choice of the types of probiotics used for PAGE. Future research should include factors not reported in previous trials (described above) and a search for other clinical probiotic strains or multi-strain mixtures that might be effective for the treatment of PAGE.

## CONCLUSIONS

In India, 2 types of probiotics (*S. boulardii* CNCM I-745 and *L. rhamnosus* GG) were well tolerated and significantly shortened both the duration of diarrhea and hospitalization stays in pediatric patients with PAGE. This is of important clinical significance. Earlier recovery and reduced hospital stays translate to less morbidity and lower healthcare costs for the system and the carers of these children.

## ACKNOWLEDGMENTS

We would like to thank Dr. Krishna C. Veligandla for his assistance in communication with coauthors and reviewing the article.

Planning and conducting the study (L.V.M., R.S., R.P.S., S.G., A.B., M.M., and B.R.), collecting and interpreting the data (L.V.M., R.S., R.P.S., S.G., A.B., M.M., and B.R.), statistical analysis (L.V.M.), drafting the article (L.V.M.), and writing the final article (L.V.M., R.S., R.P.S., S.G., A.B., M.M., B.R., and N.M.). L.V.M. accepts full responsibility for the conduct of the study. The author had access to the data and had control of the decision to publish. All authors approved the final draft submitted.

## Supplementary Material


