# Supplementary material for: Specific Probiotics for the Treatment of Pediatric Acute Gastroenteritis in India: A Systematic Review and Meta-Analysis
Source: JPGN Rep. 2021 May 27;2(3):e079. doi: 10.1097/PG9.0000000000000079 (PMC10191489; doi:10.1097/PG9.0000000000000079)
Supplement: Supplementary file 1 [file pg9-2-e079-s001.pdf]

SCD Text 1, Methods

METHODS

Search Strategy

We searched databases including PubMed, Google Scholar, and NIH registry of clinical trials (database inception to May 2020) to identify randomized controlled trials (RCTs) of probiotic use in PAGE. Our search strategy was as follows: ("probiotics" [MeSH Terms] OR "probiotics" [All Fields]) AND "acute pediatric diarrhea" [MeSH Terms] AND "clinical trials" [All Fields] and "India" [All Fields]. Additional searches were done using search terms: *Bacillus* or *Bifidobacterium* or *Escherichia* or *Enterococcus* or *Lactobacillus* or *Saccharomyces* or probiotic mixtures or VSL#3. Secondary searches of grey literature included reference lists, authors, reviews, meeting abstracts websites and clinicaltrials.gov for unpublished trials. A recursive search was also performed, using the bibliographies of all obtained articles. There were no language restrictions and articles in languages other than English were translated and reviewed.

Inclusion/exclusion criteria

Inclusion criteria included: randomized, controlled clinical trials (RCTs) in children with acute diarrhea using probiotic interventions and published in peer-reviewed journals. We included only probiotics fulfilling the standard definition (must be living microbe, of adequate dose and having efficacy for a health effect (25). This definition excludes dead or heat-killed microbes and prebiotics. As bacterial and fungal taxonomies shift over time, the most current strain designations are presented in this review and strain identification was confirmed with the original authors or the manufacturer whenever possible.

Exclusion criteria included: non-human studies, early phase 1 or 2 safety or mechanism of action studies, no control group, probiotic not well described, clinical trials in adults, chronic or persistent pediatric diarrhea, reviews and duplicate reports. Cross-over trials were excluded due to the potential for effect carry-over after short wash-out periods used in these trials. RCTs of acute pediatric diarrhea in non-Indian developing countries were excluded.

For the meta-analysis, each probiotic type was required to have at least two RCTs for each outcome assessed. We followed current recommendations requiring each type of probiotic be analyzed as a separate sub-group and not to pool dissimilar types of probiotics (16,17).

Data extraction and Assessment of Validity

The literature was searched independently by two co-authors (LM, RS). Data from all RCTs were extracted using a standardized data extraction form initially completed by one co-author (LM) and then each study was independently reviewed by at least one of the other co-authors following the standard methods for systematic reviews and meta-analysis (26,27). Any disagreements were discussed until consensus was reached. The data extracted included PICOS data: (1) patient population (pediatric, age range, country), (2) intervention (type of probiotic or controls used, daily doses, formulation, duration and follow-up times), (3) comparisons (type of control group either placebo or open, unblinded), (4) Primary outcomes, including mean duration of diarrhea, number with diarrhea resolution ('cured') by Day 3-5 and rapidity of response (stool

frequency by day 1-5), and (5) secondary outcomes; length of hospitalization and safety data. In addition, data on potential confounding factors were collected: adjunctive treatments (oral rehydration or zinc), study design (randomized, controlled trials, either double blinded or open), study quality, setting (inpatient or outpatient), urban or rural locations. For data that were required for these analyses, but not reported in the published article, we attempted to contact the author or co-authors to obtain the missing data.

Each included RCT was reviewed for quality and risk of bias and scored independently by at least two of the co-authors using standard methods (27). The risk of bias was graded (high, low or not reported) for each of six types of bias [selection bias (method of randomization and blinded allocation), performance bias (degree of blinding of study personnel and study subjects), detection bias (outcome assessor blinded), attrition bias (attrition different by group), reporting bias (*a priori* outcomes reported) and other issues (fraud or miscellaneous)] and a summary figure of bias was generated (28).

Statistical Analysis

We used the standard PRISMA (Preferred Reporting Items for Systematic reviews and Meta-analysis) guidelines for this review (see Table, Supplemental Digital Content 1, which lists checklist items) (26) [but followed more recent recommendations to account for probiotic strain specificity(16,17). This review was registered with PROSPERO (CRD42020186739). Inclusion of studies in meta-analysis also required at least two RCTs within the same probiotic strain or mixture subgroups. Statistical analysis and generation of forest plots of pooled summary estimates was performed using Stata software version 16 (Stata Corporation, College Station, Texas) with meta-analysis modules (29). Summary estimates were based on the pooled data from RCTs using the same type (strain or strains) of probiotic and sharing a common outcome measure. Dichotomous outcomes were assessed using relative risks (RR) and 95% confidence intervals (C.I.) and continuous outcomes were assessed using standard mean difference (SMD) and 95% C.I. using standard methods (27). In trials reporting only mean values and no standard deviations (SD), SD were estimated using methods recommended by Higgens et al. and when only median and interquartile range (IQR) were provided, estimates using the formulas recommended by Hozo et al. were used (27,30). Heterogeneity across trials was evaluated using the I^2 statistic, 0% indicating none and >50% indicating a high degree of heterogeneity across the trials (29). Subgroup analysis was used to explore sources of heterogeneity and was assessed with the Cochrane Q test (27). Random effects models were used for the meta-analysis if heterogeneity was found ($I^2 \geq 50\%$ for overall effect). Publication bias was assessed using funnel plots and the Egger test (29). Sequential sensitivity analysis was done to explore the extent outcomes were dependent upon a particular trial.

A priori subgroup analyses based on factors that might influence the magnitude of efficacy estimates were planned for the following: (a) daily dose ($\geq 10^{10}$/day or less 10^{10}/day) colony-forming units (cfu) of probiotic, (b) type of patient (inpatient/outpatients), (c) etiology of diarrhea (rotaviral, parasitic, bacterial), (d) time of intervention initiation from onset of diarrhea, (e) type of adjunctive treatments (oral rehydration or zinc), (f) risk of bias, (g) rural versus urban setting, (h) number requiring IV fluids during study and (i) extent of blinding.
