# Supplementary material for: Specific Probiotics for the Treatment of Pediatric Acute Gastroenteritis in India: A Systematic Review and Meta-Analysis
Source: JPGN Rep. 2021 May 27;2(3):e079. doi: 10.1097/PG9.0000000000000079 (PMC10191489; doi:10.1097/PG9.0000000000000079)
Supplement: Supplementary file 2 [file pg9-2-e079-s002.pdf]

SDC Text 2, Results.

Excluded studies

Reasons for excluding 213 studies included: reviews or meta-analyses (n=63), non-pediatric diarrheal trials (n=40), unpublished website report (n=1), phase 1 or 2 studies (n=16), phase 4 safety studies (n=2), cost studies (n=2), trials not done in India (n=81), duplicate article (n=4), non-living bacteria used (n=1), no non-probiotic control (n=2) or insufficient data in abstract (n=1). Twelve studies conducted in India which have been included in other reviews, were excluded in this review (see Table, Supplemental Digital Content 2, which lists excluded trials). Four studies were duplicates (31-34), two compared two different probiotic strains directly but had no control group (35,36), two were phase 4 safety studies (37,38), one was an unpublished meeting abstract (39), one was a website reporting pooled data from 6 sites, of which data from only 2 sites were published (40), one was a dead bacterial strain (41), and one study was in persistent diarrhea (42).

Trial characteristics

All trials were done in India. The total number of enrolled patients per trial varied from 60-662 (median=126) with a mean 168 $\pm$ 130 participants; most trials (88%) included children from 6 months to 3-5 years old, while one trial included younger children (2 weeks old) (44) and two trials included older children up to 12 years old (58) or up to 14 years old (46). Most trials enrolled inpatient children (n=17, 68%), less frequently outpatient children (n=4, 16%) or mixed inpatient and outpatients (n=2, 8%), while the type of enrolled patients were not reported in two trials (49,50). The location of the trial was set in an urban setting (10 study arms, 40%), or semi-urban (3 study arms, 12%), or mixed (1 study arm, 4%) or was not reported (11 study arms, 44%). Most trials reported low (<5%) attrition (48%), or moderate attrition (6-15%) in 40% of RCTs, while high attrition (35%) was observed in two trials (53,62) and no attrition data was provided in one trial (47).

Probiotic characteristics

A total of eight different types of probiotic interventions were identified from 22 RCTs (total of 25 treatment arms) and included in the systematic review (Table 1). Five types of single

strain probiotics were found in Indian trials: *Saccharomyces boulardii* CNCM I-745, (9 RCTs), *Lactobacillus rhamnosus* GG (6 RCTs), *Bacillus clausii* UBBC-07 (1 RCT), *L. casei* DN114001 (1 RCT), and *L. sporogenes* (1 RCT). Three types of multi-strained mixtures were also found: a 4-strain mixture *Bacillus clausii* (O/C, SIN, N/R, T, 4 RCTs), a 4 strain mixture of *Clostridium butryicum, Bacillus mesentericus, Streptococcus faecalis, L. sporogenes* (1 RCT), and an 8-strain mixture of *Lactobacillus acidophilus, L. paracasei, L. bulgaricus, L. plantarum, Bifidobacterium breve, B. infantis, B. longum and Streptococcus thermophilus*, 1 RCT). As five of the probiotic types had no confirmatory trials, these were excluded from the meta-analysis, leaving three types of probiotics with $\geq$2 RCTs per probiotic type: *S. boulardii* CNCM I-745, *L. rhamnosus* GG and the four-strain *B. clausii* mixture.

While the dose of probiotic ranged from 1×10^6 colony-forming units per day (cfu/d) to 2×10^{12} cfu/d, the daily dose used was consistent for *S. boulardii* trials (1×10^{10} cfu/d) and *B. clausii* trials (2-4 x 10^9 cfu/d), while doses for *L. rhamnosus* GG ranged from 1×10^6 to 2×10^{12} cfu/d. Probiotics were given to children in different formulations, most commonly in liquid (n=7, 28% arms), or in sachets (n=6, 24% arms), and less frequently as capsules (12%), or powder (8%), or spores (8%) or tablet (4%), while the type of formulation was not reported in 4 (16%) of treatment arms. The probiotics were usually given most commonly for 5 days (in 13 arms, 52%), or longer ranging from 6-28 days (7 arms, 28%), while two (8%) only gave the probiotic for 3-4 days, or ranged 3-10 days in one study (56) and the duration was not reported in two arms of one trial (51). The children with PAGE were usually enrolled within 48 hours from the onset of diarrhea, except for four studies ($\leq$3-4 days) (46,57,63,64). The mean time of probiotic initiation (from onset of initial diarrhea to probiotic initiation) was within 48 hours in 5 (20%) of trial arms, or within 3-4 days in 7 (28%), but no data was provided in 13 (52%) of the study arms. Of the 25 treatment arms, 12 (48%) were double-blinded when placebos were given, but 13 (52%) of the controls were open (no placebo used). Oral rehydration therapy was given to both probiotic and control groups in 20 (80%) of the arms, but was not reported in 20%. The additional use of zinc was reported in 8 (32%) of the study arms but not given or unreported in 68% of the arms.

Risk of Study Bias

The study quality varied by type of probiotic (Table 1): 75% of *B. clausii* mixtures trials, 44% of the *S. boulardii* trials and 14% of the *L. rhamnosus* GG trials were rated with high risk of study bias. Most high-risk bias ratings were due to one of three domains: Blind allocation, due to lack of a blinded person allocating the intervention was reported in 59% of the RCTs; detection bias, due to lack of a blinded outcome assessor in 53% of the trials; or performance bias, due to lack of double-blinding in 47% of the trials.

Safety

Of the 25 study arms, 11 (44%) did not collect any adverse reaction data (Table 2), but 14 (56%) did collect and report adverse event data. Of the 14 with safety data, 10 (71%) reported no adverse events were observed during the study, while 4 (29%) reported at least one child with an adverse event, but the frequency was not significantly different for probiotic compared to control groups. None of the different probiotic types were associated with significant adverse events.
