# Supplementary figures and images for: Specific Probiotics for the Treatment of Pediatric Acute Gastroenteritis in India: A Systematic Review and Meta-Analysis
Source: JPGN Rep. 2021 May 27;2(3):e079. doi: 10.1097/PG9.0000000000000079 (PMC10191489; doi:10.1097/PG9.0000000000000079)

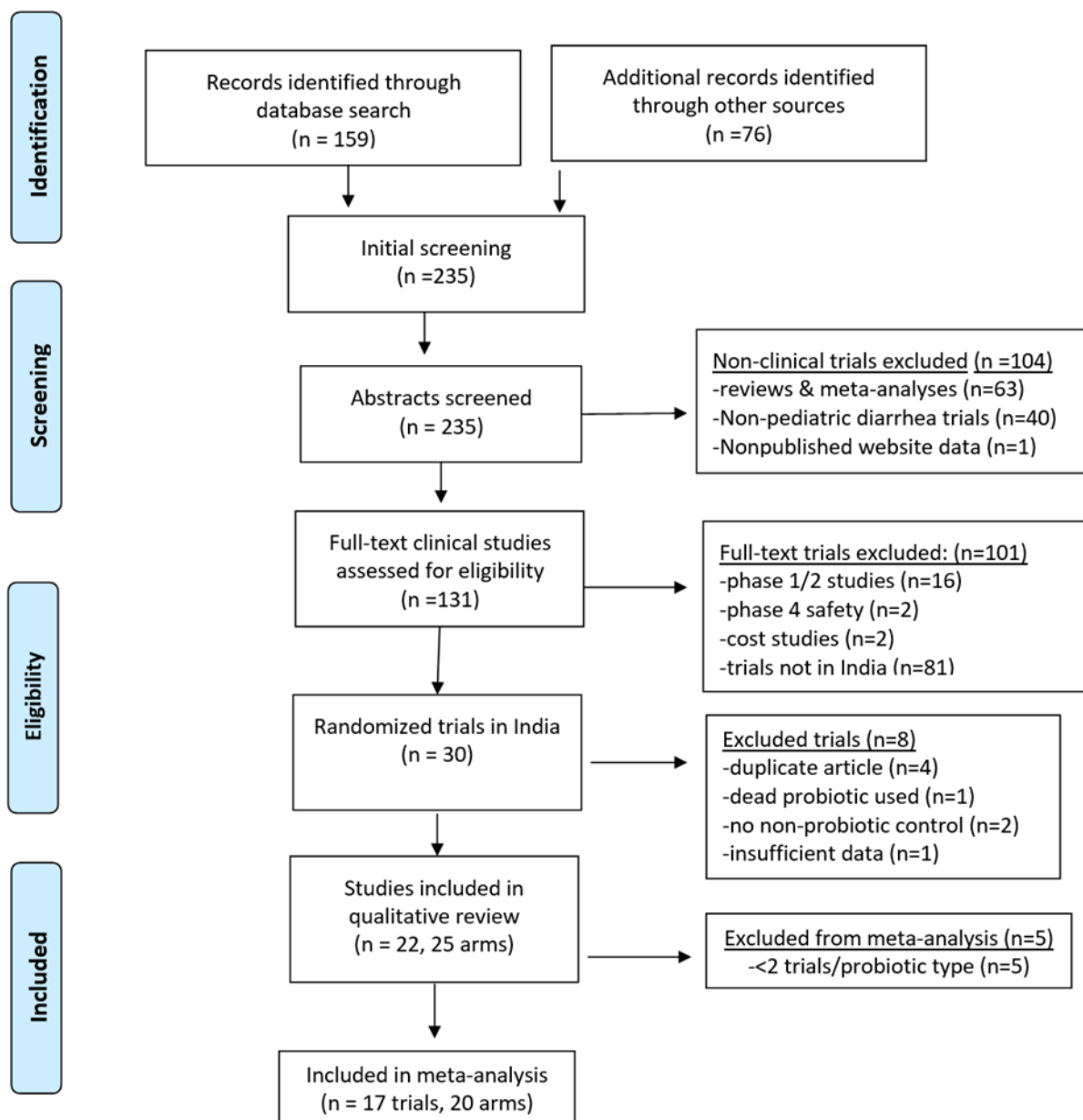

**SDC Figure 1.** PRISMA Study inclusion flow-chart

Supplement: Supplementary file 3 [file pg9-2-e079-s003.pdf]

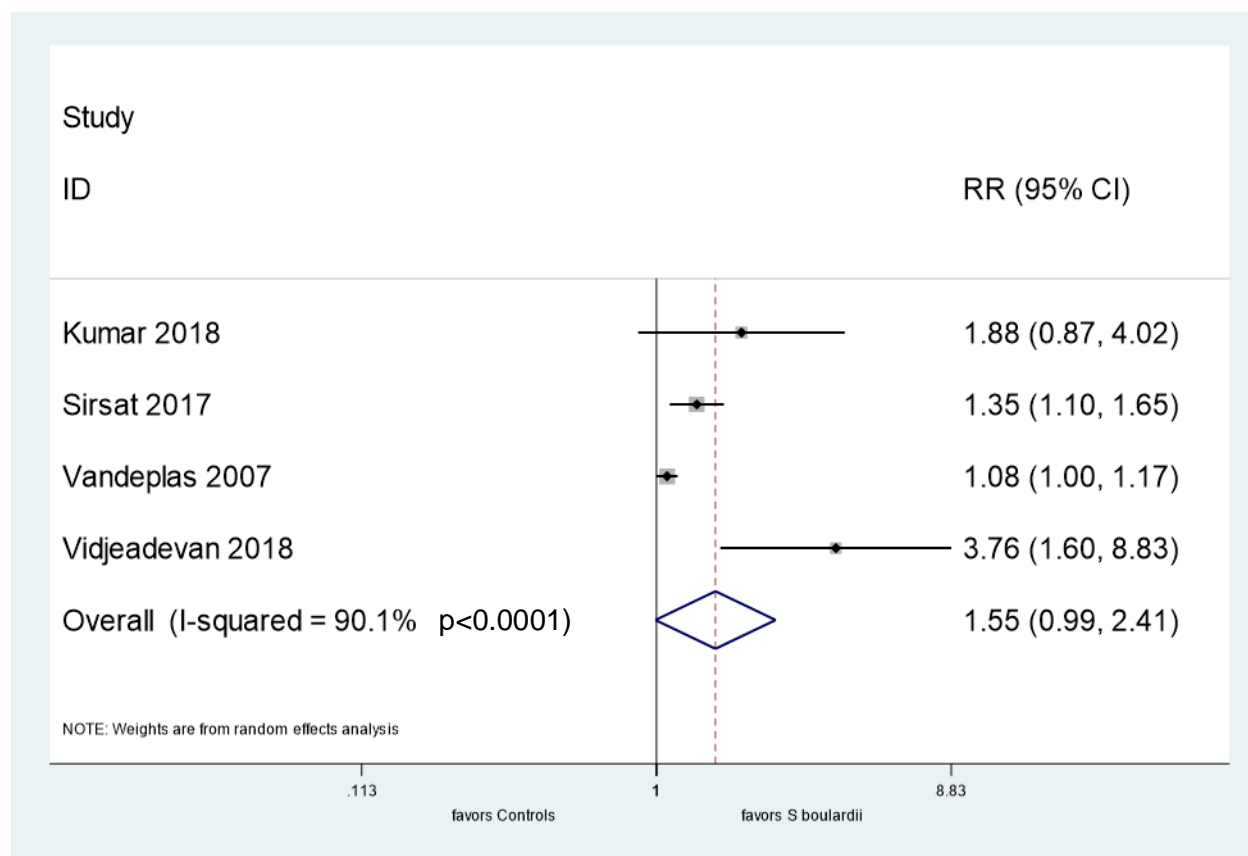

**SDC Figure 4.** Forest plot of Cured by Day 3 for *S. boulardii* CNCM I-745 trials.

Supplement: Supplementary file 6 [file pg9-2-e079-s006.pdf]

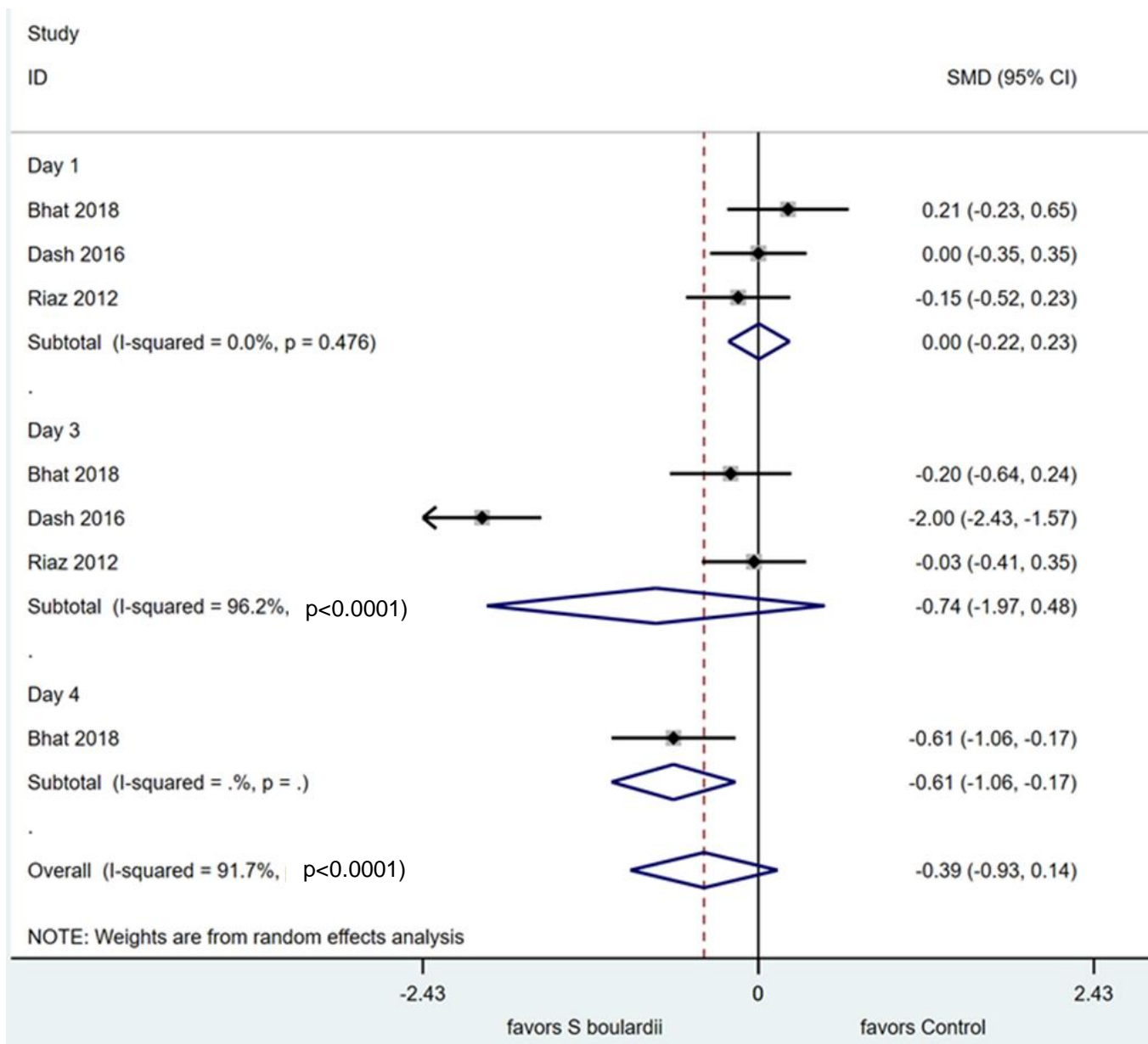

**SDC Figure 5.** Rapidity of Response. Number of Stools/day for *S. boulardii* CNCM I-745.

Supplement: Supplementary file 7 [file pg9-2-e079-s007.pdf]

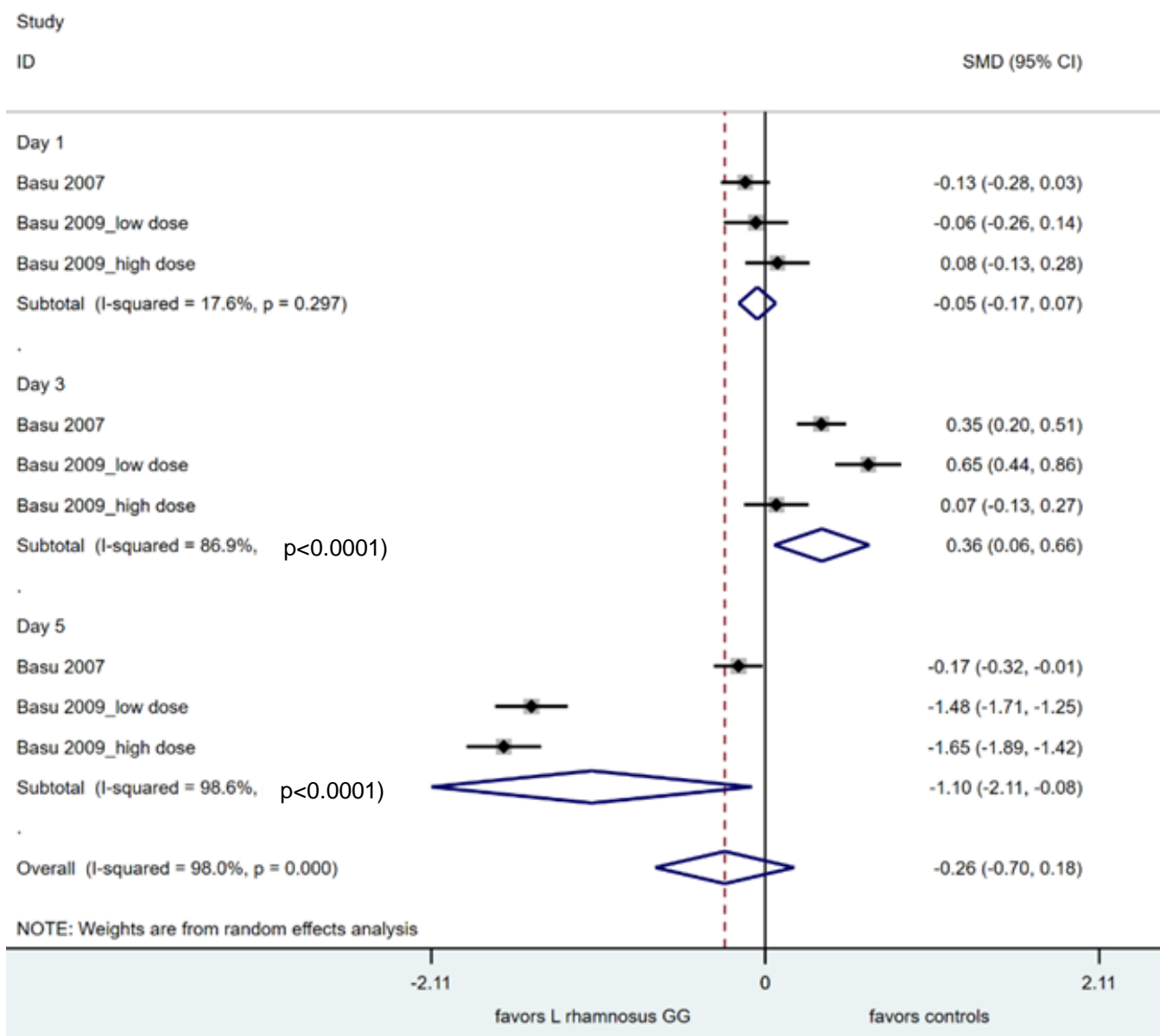

**SDC Figure 6.** Rapidity of Response. Number of Stools/day: *L. rhamnosus* GG.

Supplement: Supplementary file 8 [file pg9-2-e079-s008.pdf]

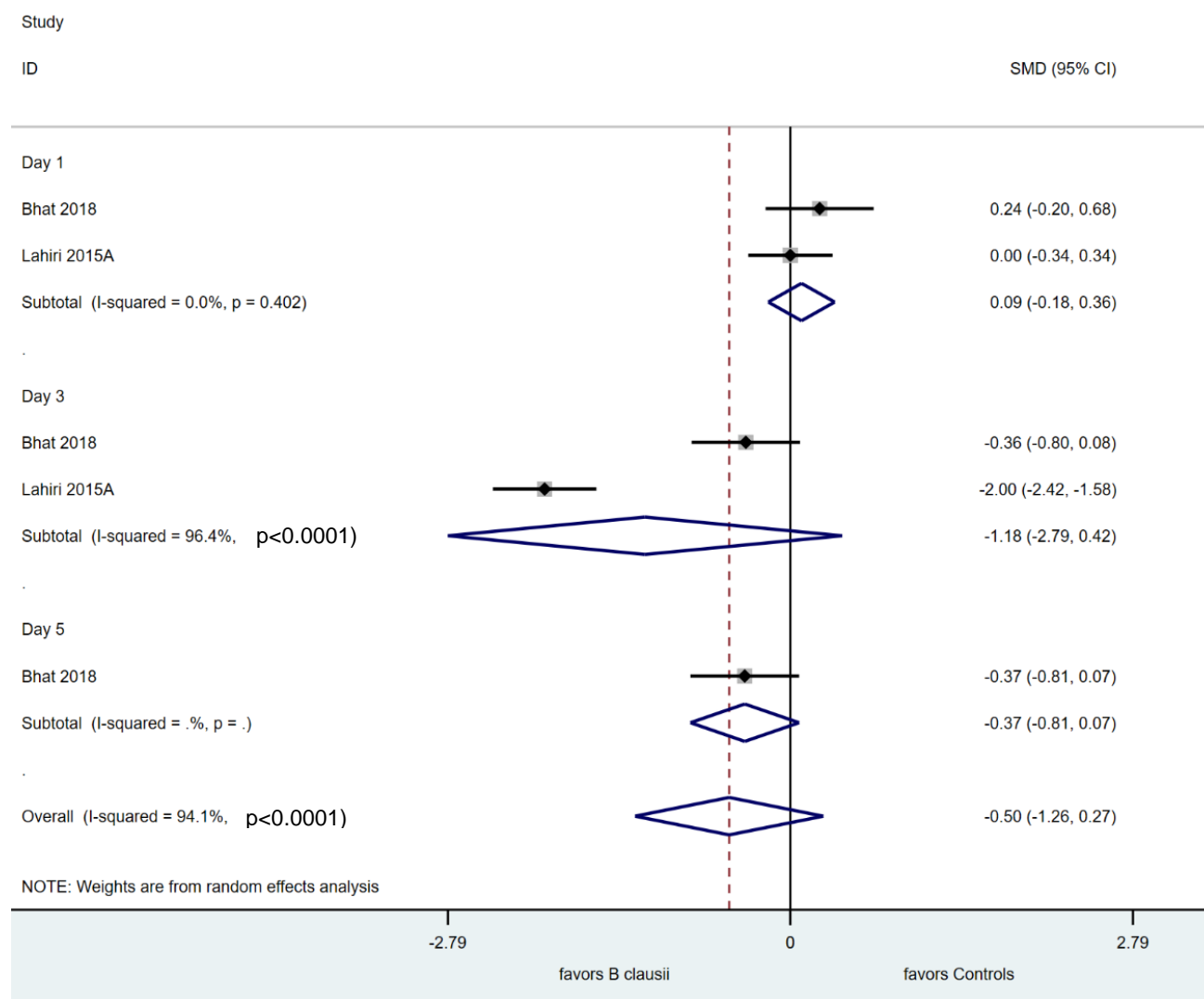

**SDC Figure 7.** Rapidity of Response. Number of Stools/day for *B. clausii* mix for Day 1,3,5.

Supplement: Supplementary file 9 [file pg9-2-e079-s009.pdf]
