# Supplementary material for: Specific Probiotics for the Treatment of Pediatric Acute Gastroenteritis in India: A Systematic Review and Meta-Analysis
Source: JPGN Rep. 2021 May 27;2(3):e079. doi: 10.1097/PG9.0000000000000079 (PMC10191489; doi:10.1097/PG9.0000000000000079)
Supplement: Supplementary file 5 [file pg9-2-e079-s005.pdf]

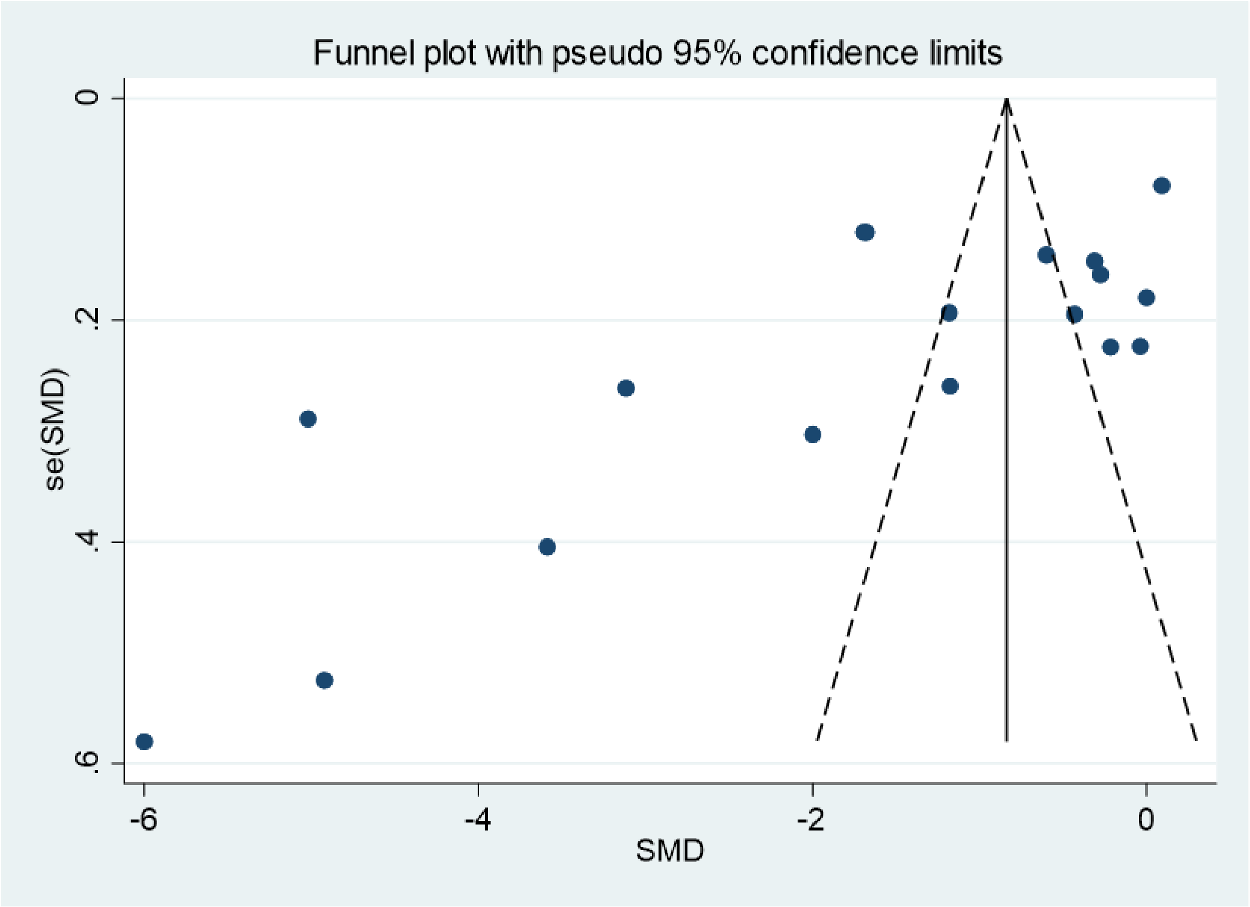

SDC Figure 3. Funnel plot for publication bias for trials of duration of diarrhea for pediatric acute gastroenteritis.
