# Supplementary material for: Specific Probiotics for the Treatment of Pediatric Acute Gastroenteritis in India: A Systematic Review and Meta-Analysis
Source: JPGN Rep. 2021 May 27;2(3):e079. doi: 10.1097/PG9.0000000000000079 (PMC10191489; doi:10.1097/PG9.0000000000000079)
Supplement: Supplementary file 12 [file pg9-2-e079-s012.pdf]

SDC Table 2. Excluded studies with probiotics for the treatment of pediatric acute gastroenteritis done in India (n=12)

Probiotic	Exclusion reason	Reference
S. boulardii vs. B. clausii	no non-probiotic controls	Vineeth S 2017 (31)
S. boulardii vs. B. clausii	no non-probiotic controls	Reddy 2013 (32)
S. boulardii I-745	duplicate of Burande 2012	Burande 2013 (33)
S. boulardii I-745	no controls, Phase 4 study	Kiran 2018 (34)
S. boulardii I-745	meeting abstract, not published, insufficient data	Sultana 2017 (35)
Bacillus clausii mix	duplicate of Lahiri 2015A	Ahmad 2018 (36)
Bacillus clausii mix	website protocol with unsupported data	Lahiri 2008 (37)
Bacillus clausii mix	Duplicate of Lahiri 2015	Lahiri 2011 (38)
Bacillus clausii mix	phase 4, no controls	Kiran 2017 (39)
L. acidophilus LA	dead (not defined probiotic)	Khanna 2005 (40)
L. casei DN114001	duplicate study of Agarwal 2002	Agarwal 2001 (41)
L. rhamnosus GG	persistent not acute diarrhea	Basu S 2007 (42)

Abbreviations: B., Bacillus; L., Lactobacillus; S., Saccharomyces
