# Supplementary material for: Specific Probiotics for the Treatment of Pediatric Acute Gastroenteritis in India: A Systematic Review and Meta-Analysis
Source: JPGN Rep. 2021 May 27;2(3):e079. doi: 10.1097/PG9.0000000000000079 (PMC10191489; doi:10.1097/PG9.0000000000000079)
Supplement: Supplementary file 13 [file pg9-2-e079-s013.pdf]

SDC Table 3. Study population characteristics for three probiotic types in randomized, controlled trials in India

**Probiotic**	**N randomized**	**Age Range**	**Type of population**	**Setting (rural/urban)**	**Attrition (%)**	**Reference**
S. boulardii CNCM I-745	80	6 mon-5 yr	inpatient	urban	0	Bhat 2018 (43)
S. boulardii CNCM I-745	72	< 5 yrs	outpatient	urban	3	Burande 2012 (44)
S. boulardii CNCM I-745	60	3 mon-5 yrs	inpatient	urban	5	Das 2016 (45)
S. boulardii CNCM I-745	126	6 mon-14 yrs	inpatient	urban	0	Dash 2016 (46)
S. boulardii CNCM I-745	100	6 mon-5 yrs	inpatient	nr	nr	Kumar 2018 (47)
S. boulardii CNCM I-745	108	3 mon-4.9 yrs	inpatient	urban	8	Riaz 2012 (48)
S. boulardii CNCM I-745	290	2 mon-5 yrs	nr	urban	0	Sirsat 2017 (49)
S. boulardii CNCM I-745	202	3 mon-3 yrs	nr	nr	7	Vandeplas 2007 (50)
S. boulardii CNCM I-745	67	6 mon-3 yrs	inpatient	nr	6	Vidjeadevan 2018 (51)
L. rhamnosus GG	200	6 mon-5 yrs	mixed (Out, ER)	nr	12.5	Aggarwal 2014 (52)
L. rhamnosus GG	100	6 mon-5 yrs	outpatient	nr	35	Agrawal 2017 (53)
L. rhamnosus GG	662	6 mon-2 yrs	inpatient	nr	2	Basu 2007 (54)
L. rhamnosus GG-low dose	329	6 mon-3 yrs	inpatient	semi-urban	6	Basu 2009 (55)
L. rhamnosus GG-high dose	329	6 mon-3 yrs	inpatient	semi-urban	6	Basu 2009 (55)
L. rhamnosus GG	229	<3 yrs	inpatient	urban	8	Misra 2009 (56)
L. rhamnosus GG	124	6 mon-5 yrs	outpatient	semi-urban	0.8	Sindhu 2014 (57)
Bacillus clausii O/C,SIN,N/R,T	80	6 mon-5 yrs	inpatient	urban	0	Bhat 2018 (43)
Bacillus clausii O/C,SIN,N/R,T	131	6 mon-12 yrs	inpatient	urban	0	Lahiri 2015 (58)
Bacillus clausii O/C,SIN,N/R,T	160	6 mon-6 yrs	inpatient	urban	0	Lahiri 2015 (59)
Bacillus clausii O/C,SIN,N/R,T	66	6 mon-3 yrs	inpatient	nr	6	Vidjeadevan 2018 (51)
Bifilac (4 strains)	80	3 mon-3 yrs	inpatient	nr	0	Narayanappa 2008 (60)
B. clausii UBBC-07	120	6 mon-5 yrs	outpatients	nr	0.8	Sudha 2019 (61)
L. casei DN114001	100	6 mon-5 yrs	mixed	mixed	35	Agarwal 2002 (62)
L. sporogenes	160	6 mon-2 yrs	inpatient	nr	7.5	Dutta 2011 (63)
8 strain mixture	230	6 mon-2 yrs	inpatient	nr	6	Dubey 2008 (64)

Notes: **L. rhamnosus** GG (ATCC 53103); **Bifilac:** 4 strain mixture: *Clostridium butyricum*, *Bacillus mesentericus*, *Streptococcus faecalis*, *Lactobacillus sporogens*, strains not reported, from author correspondence; **8 strain mixture**: *Lactobacillus plantarum* DSM24730, *Streptococcus thermophilus* DSM24731, *Bifidobacterium breve* DSM24732, *L. delbruckii ssp. bulgaricus* DSM24733, *L. paracasei* DSM24734, *Lactobacillus acidophilus* DSM24735, *B. longum* DSM24736, *B. infantis* DSM24737.

Abbreviations: ER, emergency room; nr, not reported; mon, months; out, outpatient; yrs, years.
