# Supplementary material for: Specific Probiotics for the Treatment of Pediatric Acute Gastroenteritis in India: A Systematic Review and Meta-Analysis
Source: JPGN Rep. 2021 May 27;2(3):e079. doi: 10.1097/PG9.0000000000000079 (PMC10191489; doi:10.1097/PG9.0000000000000079)
Supplement: Supplementary file 14 [file pg9-2-e079-s014.pdf]

SDC Table 4. Probiotic manufacturer and definitions of diarrhea used in trials of acute pediatric diarrhea in India.

Probiotic	Manufacturer	Brand name	Inclusion diarrhea defined	Resolution of diarrhea defined as	Reference
S. boulardii CNCM I-745	nr	nr	≥3 loose/watery stools/day	last liquid stool	Bhat 2018 (43)
S. boulardii CNCM I-745	Dr. Reddy's Labs, India	Econorm	≥3 loose/watery stools/day	2 formed stools/d	Burande 2012 (44)
S. boulardii CNCM I-745	Dr. Reddy's Labs, India	Econorm	≥3 loose/watery stools/day	last liquid stool	Das 2016 (45)
S. boulardii CNCM I-745	nr	nr	nr	nr	Dash 2016 (46)
S. boulardii CNCM I-745	Dr. Reddy's Labs, India	Econorm	≥3 loose/watery stools/day	nr	Kumar 2018 (47)
S. boulardii CNCM I-745	Dr. Reddy's Labs, India	Econorm	nr	3 formed stools/d	Riaz 2012 (48)
S. boulardii CNCM I-745	nr	nr	nr	nr	Sirsat 2017 (49)
S. boulardii CNCM I-745	nr	nr	nr	<3 stools/d	Vandeplas 2007 (50)
S. boulardii CNCM I-745	Dr. Reddy's Labs, India	Econorm	nr	nr	Vidjeadevan 2018 (51)
L. rhamnosus GG	Amerifit Brands USA	Culturelle	≥3 loose/watery stools/day	last loose stool	Aggarwal 2014 (52)
L. rhamnosus GG	nr	nr	≥3 loose/watery stools/day	nr	Agrawal 2017 (53)
L. rhamnosus GG	nr	nr	≥3 loose/watery stools/day	nr	Basu 2007 (54)
L. rhamnosus GG-low dose	nr	nr	≥3 loose/watery stools/day	nr	Basu 2009 (55)
L. rhamnosus GG-high dose	nr	nr	≥3 loose/watery stools/day	nr	Basu 2009 (55)
L. rhamnosus GG	Amerifit Brands USA	Culturelle	≥3 loose/watery stools/day	<3 stools/d	Misra 2009 (56)

L. rhamnosus GG	Health Inc. USA	Culturelle	≥3 loose/watery stools/day	nr	Sindhu 2014 (57)
Bacillus clausii O/C,SIN,N/R,T	nr	nr	≥3 loose/watery stools/day	last liquid stool	Bhat 2018 (43)
Bacillus clausii O/C,SIN,N/R,T	Sanofi Aventis, India	Enterogermnia	nr	nr	Lahiri 2015 (58)
Bacillus clausii O/C,SIN,N/R,T	Sanofi Aventis, India	Enterogermnia	nr	nr	Lahiri 2015 (59)
Bacillus clausii O/C,SIN,N/R,T	nr	Gutspert	nr	nr	Vidjeadevan 2018 (51)
Bifilac (4 strains)	nr	Bifilac	nr	nr	Narayanappa 2008 (60)
B. clausii UBBC-07	Unique Biotech Ltd., India	nr	≥3 loose/watery stools/day	nr	Sudha 2019 (61)
L. casei DN114001	Danone, France	Actimel	nr	first semi-solid or solid stool	Agarwal 2002 (62)
L. sporogenes	M/S ESKAG Pharm Pri Ltd., India	nr	nr	first formed stool	Dutta 2011 (63)
8 strain mixture	CD Pharma, India	VSL#3	nr	last liquid stool	Dubey 2008 (64)

Notes: **L. rhamnosus** GG (ATCC 53103); **Bifilac:** 4 strain mixture: *Clostridium butyricum*, *Bacillus mesentericus*, *Streptococcus faecalis*, *Lactobacillus sporogens*, strains not reported, from author correspondence;. **8 strain mixture**: *Lactobacillus plantarum* DSM24730, *Streptococcus thermophilus* DSM24731, *Bifidobacterium breve* DSM24732, *L. delbruckii ssp. bulgaricus* DSM24733, *L. paracasei* DSM24734, *Lactobacillus acidophilus* DSM24735, *B. longum* DSM24736, *B. infantis* DSM24737.

Abbreviations: d, day; nr, not reported
