# Supplementary material for: Specific Probiotics for the Treatment of Pediatric Acute Gastroenteritis in India: A Systematic Review and Meta-Analysis
Source: JPGN Rep. 2021 May 27;2(3):e079. doi: 10.1097/PG9.0000000000000079 (PMC10191489; doi:10.1097/PG9.0000000000000079)
Supplement: Supplementary file 15 [file pg9-2-e079-s015.pdf]

**SDC Table 5**. Sub-group analysis for probiotic efficacy for the reduction of the duration of diarrhea.

| **Sub-groups** | ***S. boulardii* CNCM I-745** | | | ***L. rhamnosus* GG** | | | ***B. clausii* O/C,SIN, N/R, T** | | |
|---|---|---|---|---|---|---|---|---|---|
| | **SMD (95% C.I.** | **P value** | **$I^2$** | **SMD (95% C.I.** | **P-value** | **$I^2$** | **SMD (95% C.I.** | **P-value** | **$I^2$** |
| **Population**: | | | | | | | | | |
| inpatient/mixed | -1.99 (-3.09, -0.90) | <0.001 | 97.1% | -0.97 (-1.94, 0.00) | 0.050 | 98.7% | -1.39 (-2.74, -0.04) | <0.001 | 97.3% |
| outpatient | -1.18 (-1.69, -0.67) | <0.001 | nd | -2.86 (-6.34, 0.61) | 0.11 | 99.2% | na | na | na |
| **Daily probiotic dose:** | | | | | | | | | |
| Low dose | na | na | na | -0.24 (-0.92, 0.43) | 0.48 | 94.5% | -2.00 (-2.59, -1.41) | <0.001 | nd |
| High dose | -1.85 (-2.80, -0.91) | <0.001 | 96.5% | -2.36 (-3.57, -1.15) | <0.001 | 98.4% | -1.19 (-2.85, 0.47) | 0.16 | 97.9% |
| **Risk of Bias:** | | | | | | | | | |
| High risk | -0.62 (-1.74, 0.51) | 0.28 | 93.3% | -3.59 (-4.38, -2.79) | <0.001 | nd | -1.19 (-2.85, 0.47) | 0.16 | 97.9% |
| Low risk | -2.45 (-3.87, -1.03) | 0.001 | 97.5% | -1.46 (-2.48, -0.44) | 0.005 | 98.9% | na | na | na |
| **Adequate blinding:** | | | | | | | | | |
| Open controls | -1.98 (-3.42, -0.53) | 0.007 | 96.8% | -4.33 (-5.73, -2.93) | <0.001 | 87.9% | -1.39 (-2.74, -0.04) | 0.04 | 97.3% |
| Placebo | -1.76 (-3.33, -0.20) | 0.03 | 97.2% | -0.78 (-1.61, 0.05) | 0.07 | 98.4% | na | na | na |
| **Zinc given:** | | | | | | | | | |
| Yes | -2.05 (-3.36, -0.75) | 0.002 | 96.4% | -5.02 (-5.59, -4.45) | <0.001 | nd | -1.79 (-3.63, 0.05) | 0.06 | 97.9% |
| No | -1.65 (-3.39, 0.08) | 0.062 | 97.4% | -1.20 (-2.03, -0.37) | 0.005 | 98.3% | na | na | na |

**Notes**: Dose, low dose ($<10^{10}$/day for *S. boulardii* and *L. rhamnosus* GG or 2 x $10^9$/d for *B. clausii* mix); high dose ($\geq 10^{10}$/day for *S. boulardii* and *L. rhamnosus* GG or 4 x $10^9$/day for *B. clausii* mix).

**Abbreviations**: B., *Bacillus*; C.I., confidence interval; HDI, human development index (0.6, medium; 0.8 high); $I^2$, percent heterogeneity L., *Lactobacillus*; na, not applicable as no trials in category or missing data; nd, not determined as only one trial in category; S., *Saccharomyces*
