# Supplementary material for: Specific Probiotics for the Treatment of Pediatric Acute Gastroenteritis in India: A Systematic Review and Meta-Analysis
Source: JPGN Rep. 2021 May 27;2(3):e079. doi: 10.1097/PG9.0000000000000079 (PMC10191489; doi:10.1097/PG9.0000000000000079)
Supplement: Supplementary file 16 [file pg9-2-e079-s016.pdf]

SDC Table 6. Duration of diarrhea outcomes for rotavirus positive children in trials done in India by different probiotics.

	Probiotic		Controls		Reference
**Probiotic**	**No.**	**Rotavirus + Duration diarrhea (mean + std dev hours)**	**No.**	**Rotavirus + Duration diarrhea (mean + std dev hours)**	
S. boulardii CNCM I-745		nr		nr	Bhat 2018 (43)
S. boulardii CNCM I-745		nr			Burande 2012 (44)
S. boulardii CNCM I-745	30	60 + 4	30	89 + 7	Das 2016 (45)
S. boulardii CNCM I-745		nr		nr	Dash 2016 (46)
S. boulardii CNCM I-745		nr		nr	Kumar 2018 (47)
S. boulardii CNCM I-745		nr		nr	Riaz 2012 (48)
S. boulardii CNCM I-745	88	60/88 (64%)*	77	30/77 (39%)	Sirsat 2017 (49)
S. boulardii CNCM I-745		nr		nr	Vandeplas 2007 (50)
S. boulardii CNCM I-745		nr		nr	Vidjeadevan 2018 (51)
L. rhamnosus GG	21	60 + 3	20	84 + 4.5	Aggarwal 2014 (52)
L. rhamnosus GG	nr	60 + 3	nr	84 + 4.5	Agrawal 2017 (53)
L. rhamnosus GG		nr		nr	Basu 2007 (54)
L. rhamnosus GG-low dose		nr		nr	Basu 2009 (55)
L. rhamnosus GG-high dose		nr		nr	Basu 2009 (55)
L. rhamnosus GG		nr		nr	Misra 2009 (56)
L. rhamnosus GG	45	4 + 0.5	37	4 + 0.7	Sindhu 2014 (57)
Bacillus clausii O/C,SIN,N/R,T		nr		nr	Bhat 2018 (43)
Bacillus clausii O/C,SIN,N/R,T		nr		nr	Lahiri 2015 (58)
Bacillus clausii O/C,SIN,N/R,T		nr		nr	Lahiri 2015 (59)
Bacillus clausii O/C,SIN,N/R,T		nr		nr	Vidjeadevan 2018 (51)
Bifilac (4 strains)	40	104.4 + 28.8	40	130.8 + 40.6	Narayanappa 2008 (60)
B. clausii UBBC-07		nr		nr	Sudha 2019 (61)
L. casei DN114001		nr		nr	Agarwal 2002 (62)
L. sporogenes	25	28.7 + 18.8	26	32.5 + 17.8	Dutta 2011 (63)
8 strain mixture		nr		nr	Dubey 2008 (64)

Notes: L. rhamnosus GG (ATCC 53103); **Bifilac:** 4 strain mixture: *Clostridium butyricum, Bacillus mesentericus, Streptococcus faecalis, Lactobacillus sporogens*, strains not reported, from author correspondence; **8 strain mixture**: *Lactobacillus plantarum* DSM24730, *Streptococcus thermophilus* DSM24731, *Bifidobacterium breve* DSM24732, *L. delbruckii ssp. bulgaricus* DSM24733, *L. paracasei* DSM24734, *Lactobacillus acidophilus* DSM24735, *B. longum* DSM24736, *B. infantis* DSM24737.

Abbreviations: B., *Bacillus*; L., *Lactobacillus*; nr, not reported; S., *Saccharomyces*; std dev, standard deviation
