# Supplementary material for: Specific Probiotics for the Treatment of Pediatric Acute Gastroenteritis in India: A Systematic Review and Meta-Analysis
Source: JPGN Rep. 2021 May 27;2(3):e079. doi: 10.1097/PG9.0000000000000079 (PMC10191489; doi:10.1097/PG9.0000000000000079)
Supplement: Supplementary file 17 [file pg9-2-e079-s017.pdf]

SDC Table 7. Number of daily stools/day for Baseline, Day 3 and Day 5 of treatment for different probiotics.

Daily frequency of stools/day (mean and standard deviation)									
	Day 1				Day 3		Day 4-5		
**Probiotic**	**No.**	**Probiotic**	**No.**	**Controls**	**Probiotic**	**Controls**	**Probiotic**	**Controls**	**Reference**
S. boulardii CNCM I-745	40	6.9 ± 2.8	40	6.3 ± 2.8	2.6 ± 1.3	3 ± 2.5	0.3 ± 0.8	1.3 ± 2.1	Bhat 2018 (43)
S. boulardii CNCM I-745	na	nr	na	nr	nr	nr	nr	nr	Burande 2012 (44)
S. boulardii CNCM I-745	na	nr	na	nr	nr	nr	nr	nr	Das 2016 (45)
S. boulardii CNCM I-745	64	8 ± nr	62	8 ± nr	1 ± nr	2 ± nr	nr	nr	Dash 2016 (46)
S. boulardii CNCM I-745	na	nr	na	nr	nr	nr	nr	nr	Kumar 2018 (47)
S. boulardii CNCM I-745	54	27 ± 19.2	54	30 ± 22.2	15.3 ± 11.7	15.0 ± 13.4	nr	nr	Riaz 2012 (48)
S. boulardii CNCM I-745	na	nr	na	nr	nr	nr	nr	nr	Sirsat 2017 (49)
S. boulardii CNCM I-745	na	nr	na	nr	nr	nr	nr	nr	Vandeplas 2007 (50)
S. boulardii CNCM I-745	na	nr	na	nr	nr	nr	nr	nr	Vidjeadevan 2018 (51)
L. rhamnosus GG	na	nr	na	nr	nr	nr	nr	nr	Aggarwal 2014 (52)
L. rhamnosus GG	na	nr	na	nr	nr	nr	nr	nr	Agrawal 2017 (53)
L. rhamnosus GG	323	22.4 ± 5.3	323	24.1 ± 5.8	18.4 ± 3.2	17.3 ± 3.0	6.4 ± 2.6	6.8 ± 2.1	Basu 2007 (54)
L. rhamnosus GG-low dose	188	24.8 ± 5.4	185	25.1 ± 4.7	21.7 ± 5.7	21.7 ± 5.4	3.2 ± 2.6	10.3 ± 6.3	Basu 2009 (55)
L. rhamnosus GG-high dose	186	25.5 ± 5.6	185	25.1 ± 4.7	22.1 ± 6.0	21.7 ± 5.4	3.3 ± 2.6	10.3 ± 6.3	Basu 2009 (55)
L. rhamnosus GG	105	nr	105	nr	2.03 ± nr	2.1 ± nr	2.6 ± nr	2.6 ± nr	Misra 2009 (56)
L. rhamnosus GG	na	nr	na	nr	nr	nr	nr	nr	Sindhu 2014 (57)
Bacillus clausii O/C,SIN,N/R,T	40	6.9 ± 2.2	40	6.3 ± 2.8	2.3 ± 1.1	3 ± 2.5	0.1 ± 0.3	0.3 ± 0.7	Bhat 2018 (43)
Bacillus clausii O/C,SIN,N/R,T	69	4 ± nr	62	4 ± nr	1 ± nr	2 ± nr	nr	nr	Lahiri 2015 (58)
Bacillus clausii O/C,SIN,N/R,T	na	nr	na	nr	nr	nr	nr	nr	Lahiri 2015 (59)
Bacillus clausii O/C,SIN,N/R,T	na	nr	na	nr	nr	nr	nr	nr	Vidjeadevan 2018 (51)
Bifilac (4 strains)	na	nr	na	nr	nr	nr	nr	nr	Narayanappa 2008 (60)
B. clausii UBBC-07	59	8.7 ± 3.4	60	8.5 ± 3.2	5.5 ± 1.0	5.9 ± 1.1	3.6 ± 0.1	3.6 ± 0.1	Sudha 2019 (61)
L. casei DN114001	na	nr	na	nr	nr	nr	nr	nr	Agarwal 2002 (62)
L. sporogenes	na	nr	na	nr	nr	nr	nr	nr	Dutta 2011 (63)
8 strain mixture	113	13 ± nr	111	14 ± nr	5.1 ± nr	7.0 ± nr	nr	nr	Dubey 2008 (64)

Notes: **L. rhamnosus** GG (ATCC 53103); **Bifilac:** 4 strain mixture: *Clostridium butyricum*, *Bacillus mesentericus*, *Streptococcus faecalis*, *Lactobacillus sporogens*, strains not reported, from author correspondence;. **8 strain mixture**: *Lactobacillus plantarum* DSM24730, *Streptococcus thermophilus* DSM24731, *Bifidobacterium breve* DSM24732, *L. delbruckii ssp. bulgaricus* DSM24733, *L. paracasei* DSM24734, *Lactobacillus acidophilus* DSM24735, *B. longum* DSM24736, *B. infantis* DSM24737.

Abbreviations: na, not applicable as outcome not reported in paper; nr, not reported; mon, months; yrs, years
