# Supplementary material for: Specific Probiotics for the Treatment of Pediatric Acute Gastroenteritis in India: A Systematic Review and Meta-Analysis
Source: JPGN Rep. 2021 May 27;2(3):e079. doi: 10.1097/PG9.0000000000000079 (PMC10191489; doi:10.1097/PG9.0000000000000079)
Supplement: Supplementary file 18 [file pg9-2-e079-s018.pdf]

SDC Table 8. Length-of-hospital stay outcomes for inpatient pediatric patients in trials done in India for different probiotics

	Probiotic		Controls		Reference
**Probiotic**	**No.**	**Length of stay (mean ± std dev days)**	**No.**	**Length of stay (mean ± std dev days)**	
S. boulardii CNCM I-745	40	2.7 ± 0.4	40	3.4 ± 1.1	Bhat 2018 (43)
S. boulardii CNCM I-745		nr		nr	Burande 2012 (44)
S. boulardii CNCM I-745	30	3.1 ± 0.3	30	3.8 ± 0.3	Das 2016 (45)
S. boulardii CNCM I-745	64	2.7 ± nr	62	4.8 ± nr	Dash 2016 (46)
S. boulardii CNCM I-745		nr		nr	Kumar 2018 (47)
S. boulardii CNCM I-745		nr		nr	Riaz 2012 (48)
S. boulardii CNCM I-745		nr		nr	Sirsat 2017 (49)
S. boulardii CNCM I-745		nr		nr	Vandeplas 2007 (50)
S. boulardii CNCM I-745	34	3.4 ± 1.0	33	3.3 ± 1.1	Vidjeadevan 2018 (51)
L. rhamnosus GG	100	3.3 ± 0.6	100	3.8 ± 0.6	Aggarwal 2014 (52)
L. rhamnosus GG	32	3.3 ± 0.5	33	3.7 ± 0.6	Agrawal 2017 (53)
L. rhamnosus GG	323	9.3 ± 1.3	323	9.2 ± 1.3	Basu 2007 (54)
L. rhamnosus GG-low dose	188	6.2 ± 1.2	185	9.7 ± 2.1	Basu 2009 (55)
L. rhamnosus GG-high dose	186	6.2 ± 1.1	185	9.7 ± 2.1	Basu 2009 (55)
L. rhamnosus GG		nr		nr	Misra 2009 (56)
L. rhamnosus GG		nr		nr	Sindhu 2014 (57)
Bacillus clausii O/C,SIN,N/R,T	40	3.2 ± 0.7	40	3.4 ± 1.1	Bhat 2018 (43)
Bacillus clausii O/C,SIN,N/R,T	69	2.8 ± nr	62	4.3 ± nr	Lahiri 2015 (58)
Bacillus clausii O/C,SIN,N/R,T		nr		nr	Lahiri 2015 (59)
Bacillus clausii O/C,SIN,N/R,T	33	3.1 ± 0.9	33	3.3 ± 1.1	Vidjeadevan 2018 (51)
Bifilac (4 strains)		nr		nr	Narayanappa 2008 (60)
B. clausii UBBC-07		nr		nr	Sudha 2019 (61)
L. casei DN114001		nr		nr	Agarwal 2002 (62)
L. sporogenes		nr		nr	Dutta 2011 (63)
8 strain mixture		nr		nr	Dubey 2008 (64)

Notes: **L. rhamnosus** GG (ATCC 53103); **Bifilac:** 4 strain mixture: *Clostridium butyricum*, *Bacillus mesentericus*, *Streptococcus faecalis*, *Lactobacillus sporogens*, strains not reported, from author correspondence; **8 strain mixture**: *Lactobacillus plantarum* DSM24730, *Streptococcus thermophilus* DSM24731, *Bifidobacterium breve* DSM24732, *L. delbruckii ssp. bulgaricus* DSM24733, *L. paracasei* DSM24734, *Lactobacillus acidophilus* DSM24735, *B. longum* DSM24736, *B. infantis* DSM24737.

Abbreviations: nr, not reported in paper; std dev, standard deviation
